# Impact of elevation and slope aspect on floristic composition in wadi Elkor, Sarawat Mountain, Saudi Arabia

**DOI:** 10.1038/s41598-021-95450-4

**Published:** 2021-08-09

**Authors:** Mohamed A. Fadl, Hatim M. Al-Yasi, Emad A. Alsherif

**Affiliations:** 1 Department of Biology, College of Science, Taif University, P.O. Box 11099, Taif, 21944 Saudi Arabia; 2 Department of Botany and Microbiology, Faculty of Science, Beni-Suef University, Beni Suef, Egypt; 3 Department of Biology, College of Science and Arts, University of Jeddah, Jeddah, Saudi Arabia

**Keywords:** Biodiversity, Biogeography, Ecology, Ecology

## Abstract

The current research was carried out in Wadi Elkor, one of the Sarawat Mountains regions, which is a special location from an environmental standpoint and one of the only places in Saudi Arabia where a range of Palaearctic flora co-exists with Afrotropical species. The study aimed to determine the floristic composition as well as the effects of slope aspect and elevation on species, life forms, and phytogeographical elements distribution. The study area is located in Wadi Elkor, a valley in the Sarawat Mountains that cuts off the Al-Hada escarpment, 47 km southeast of Makkah City, Saudi Arabia. We conducted the research at three different locations, each with a different elevation and slope aspect. Based on floristic composition, Ward classification moreover Jaccard comparisons were performed. A total of 189 species was discovered, divided into 131 genera and 43 families. The current study identified *Argyrolobium rarum* Dumme as a new vascular plant in Saudi Arabia's terrestrial flora. In the current study, the Gramineae, Leguminosae, and Compositae families contributed 29% of the total plant species, whereas 14 families were represented by one species each. *Solanum* was the most numerous genus, with seven species, followed by *Acacia* and *Pulicaria*, each with six species, while *Commicarpos* and *Euphorbia*, each with four species. At an elevation of 1060 m above sea level, the north facing slope had the most plants, genera, and families. Therophytes had the most species, accounting for 44%, followed by Chamaephytes, which accounted for 26%. Hemicryptophytes accounted for 12% of the total, while phanerophytes accounted for 10%. In the studied area, the bioregional Saharo-Sindian and Sudano-Zambesian groups were the most well-represented (41%). The floristic composition, as well as the distribution of life forms and phytogeographical components, were found to be significantly affected by the elevation and slope aspects. The study showed that slope aspect and elevation both affected the distribution of plant species, with elevation being the most influential of the two variables.

## Introduction

Native plant diversity is widely regarded as an integral component of terrestrial ecosystems^1^. Its main role is to provide ecological viability in a given area, but it also stabilizes slopes, buffers weather, improve soils, and offers habitats for wild fauna^2^. Saudi Arabia is a large region of arid land that covers more than 2 million km^2^. The country covers the major part of the Arabian Peninsula and contains many ecosystems differing in plant diversity^3–5^. The components of Saudi`s flora come from Asia, Africa, and the Mediterranean and include medicinal plants as well as important genetic sources for crops and fodders^4,6,7^.

Mountains, according to Körner^8^, have a major impact on global trends in species richness. Mountains are beneficial to many endemic species and preserve plant populations because of their special climatic conditions and diversity of ecosystems, which differ from the conditions in their surroundings^9,10,10^. Life-form diversity is normally correlated with climatic heterogeneity^11^ and decreases with increasing elevations^12,13^. This is generally explained as a result of changing circumstance^14^, as the elevation rises, the climatic conditions become colder. It has been repeatedly shown that life-form spectra (proportion of species belonging to individual life forms) can be predicted from particular climate properties, for any continent, biogeographic region, and elevation^14,15^. Conversely, the life-form spectrum gives basic climatic information^16–20^.

The Asir Mountains of Saudi Arabia form a continuous chain of escarpments, which extend from the Yemen border to Taif, running parallel to the Red Sea. Osman et al.^21^ recorded that southwestern and northwestern Saudi Arabia was densely vegetated and contained almost 70% of Saudi’s flora. The difference in species diversity of particular regions or habitats can be attributed to many ecological gradients^22–24^. The elevational gradient in particular is one of the critical factors shaping the local patterns of the floristic diversity^25–32^. Previous studies also confirmed that the contrast between two opposite sides, north and south, is considered one of the factors affecting floral distribution due to the differences in the received solar radiation (e.g., the study of Kutiel and Lavee^33^ in the Middle East, Cantlon^34^ in North America, Vetaas^35^ in East Africa, and Kirkpatrick et al.^36^ in Australia). The current study area is ecologically unique and lies between Eurasia and Africa, which contains many Afrotropical species^21^. Its difficult topography and relative inaccessibility have led to a paucity of information about the effect of the slope aspect and elevation on species distribution. This study aims to identify the floristic composition and the effects of slope aspect and elevation on the distribution of species, life forms, and phytogeographical elements where a range of Palaearctic flora co-exists with Afrotropical plants.

## Material and methods

### Study area

**Figure 1:**
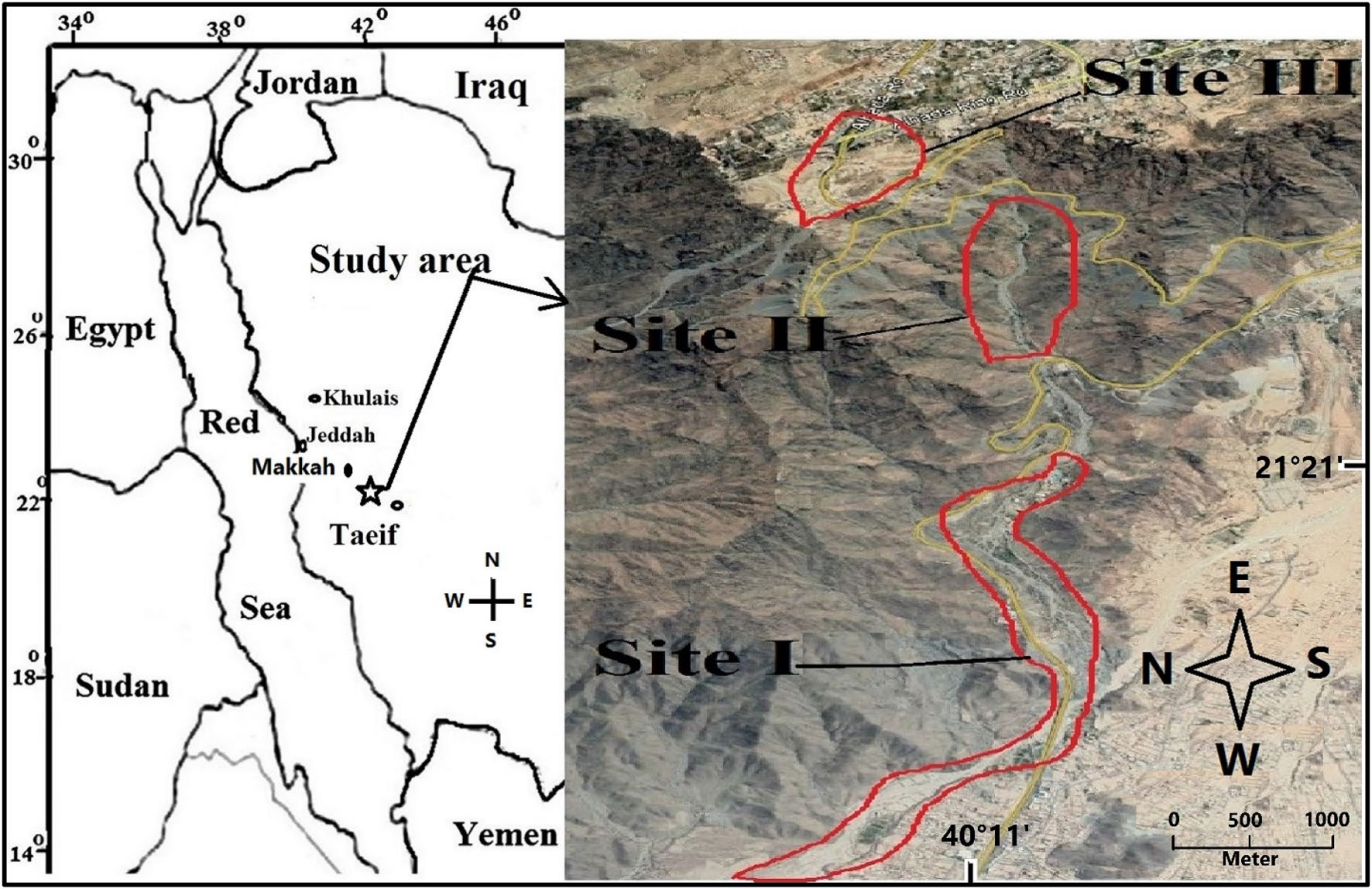
Diagram of the studied three sites (Image modified from the URL: https://www.google.com/maps/@21.385676,40.2515776,11109m/data=!3m1!1e3).

The study area (Fig. 1) is located at the beginning of the Al-Hada highlands in the Sarawat mountain, 47 km southeast of Makkah City, Saudi Arabia (from 21°21′39.21″N 40°15′47.69″E to 21°21′24.08″N 40°14′21.93″E). The foothills and highland slopes are created mainly of resistant, coarse, pink granite, mixed with grey diorite and granodiorite^37^. The rocks are broadly exposed, steep-faced, with little soil cover and a little bit covered with vegetation that is fundamentally constrained to crevices or small depressions in which fine sediments have aggregated into pockets. Large boulders, gravel, and small stones are present in the steep runnels. The climate is arid with 181 mm of the 30-year average annual rainfall^38^. The rainy season is between April and November, and the mean annual temperature is 22.8 °C, with the coldest mean temperatures (15 °C) in January and the warmest (29 °C) in July (Table 1).

**Table 1:** Climate data for the two cities between which the study area is located.

	Average high temperature (°C)		Average low temperature (°C)		Annual average humidity (%)		Annual average rainfall (mm)	
	Makkah	Taif	Makkah	Taif	Makkah	Taif	Makkah	Taif
January	30.5	22.6	18.8	8.4	58	61	20.8	9.9
February	31.7	24.6	19.1	9.9	54	54	3	1.6
March	34.9	27.3	21.1	12.5	48	47	5.5	15.1
April	38.7	30.1	24.5	15.5	43	47	10.3	35.7
May	42	33.5	27.6	19.1	36	38	1.2	35.3
June	43.8	35.8	28.6	22.3	33	25	0	3.9
July	43	35.2	29.1	23.2	34	27	1.4	2.1
August	42.8	35.7	29.5	23.6	39	31	5	17.9
September	42.8	34.8	28.9	20.8	45	33	5.4	10.6
October	40.1	30.7	25.9	15.8	50	42	14.5	14.6
November	35.2	26.7	23	12.3	58	56	22.6	25
December	32	23.8	20.3	9.5	59	61	22.1	7.6

### Sample collection

An extensive survey was carried out during the period of March 2019 to March 2020. 'Three sites were studied in the valley (Fig. 1): Site I is located in the Al-Hada escarpment and is connected to the main bed of Wadi Al-Numan. At an altitude of 750 m, it is the lowest of the three sites. Site II is located on an escarpment of Al-Hada that ranges in elevation from 1060 to 1240 m above sea level with north and south facing aspects. Site III is located at the beginning of Al-Hada mountain, at an altitude of 1830 m. The near-vertical rise in elevation from 1240 to 1830 m meant it was impossible to study vegetation in this elevation belt. To study the effect of slope, aspect and elevation on growth, Site II was divided into six belts, with three different height classes assigned to both sides. The plant samples were collected following the guidelines and legislation of both the Environmental Affairs Agency and the Wildlife Authority in the Taif Governorate. Plant samples were collected from 34 stands (five stands for each belt of elevation, except at 1800 m a.s.l. with four stands), which represented the different habitats of the study area. These are usually placed perpendicular to the slope. In each stand, we marked five 10 m × 20 m plots, each separated by 30 m. We purposefully located the plots in areas selected with similar vegetation because of the various habitats in some elevational belts. The study area is not a nature reserve and it is allowed to collect plant samples from it for study. Species to genera ratios in the studied belts were determined and we visually estimated the dominant and codominant species in each site. The lead author, Fadl M.A., identified and named the gathered plant species according to Migahid^39^, Collenette^40^, and Chaudhary^41^. The collected samples were deposited in the Herbarium of the Biology Department at Taif University and the ID numbers of the voucher specimens are presented in Supplementary Information: Appendix 1. The life forms of the identified species were determined relying on the location of the regenerative buds and the parts that were shed during the undesirable season^42^. The chorology of the studied species was determined according to the method of Wickens^43^ and Zohary^44^.

### Soil analysis

Soil texture was determined by the hydrometer method, which provided quantitative data on the percentage of sand, silt, and clay^45^. Soil salinity and pH were determined in a saturated soil paste extract with conductivity and pH meters, respectively^46^. Organic matter was determined by the Walkley Black method^45^, while total nitrogen was calculated according to the method of Bremmer^46^.

### Statistical analyses

Floristic similarities among elevation belts were assessed by performing a hierarchical classification analysis based on presence/absence data with Wards’ (minimum variance) method and Euclidean distances as a dissimilarity measure^47^. The analysis was undertaken using the Statistica statistical software package ver. 8 (StatSoft, Inc., Tulsa, OK, USA). The Jaccard similarity index was applied to evaluate ß-diversity/similarity among stands according to the following formula: Jaccard Index = (the number in both sites)/(the number in either site) * 10), which is based on the presence/absence of species^48^.

## Results

### Soil characters

The results of soil character showed slight differences in soil texture and electrical conductivity of all sites, including all elevation belts of Site II (Table 2). Soil alkalinity (pH) ranged between 7.4 and 7.8. Total nitrogen, organic carbon, and moisture, of site II, recorded higher values than the other two sites by 27%, 37% and 96%, respectively, with maximum values on the north facing slope at an elevation of 1060 m.a.s.l. At the same time, soil characters in site II showed that the north facing slope exhibited higher values of total nitrogen, organic carbon, and moisture than the south facing slope at all elevation belts by about 35%, 15% and 200%, respectively.

**Table 2:** Soil parameters registered along with the three sites of the studies area, EC, electrical conductivity; OM, organic matter; N, total nitrogen.

	Site 1 (Lower)	Site 2 (Middle)						Site 3 (Upper)
		1060 m.a.s.l		1160 m.a.s.l		1240 m.a.s.l		
		North Face	South Face	North Face	South Face	North Face	South Face	
Sand (%)	93.5 ± 1.5	95 ± 0.8	94.8 ± 1.5	95.2 ± 1.3	94.8 ± 1.6	96.1 ± 1.5	96.2 ± 2	97 ± 1.1
Silt (%)	4.1 ± 0.1	2.3 ± 0.1	2.1 ± 0.2	2.4 ± 0.1	3.1 ± 0.2	2.3 ± 0.1	2.4 ± 0.1	1.9 ± 0.2
Clay (%)	2.4 ± 0.1	2.7 ± 0.1	3.1 ± 0.1	2.4 ± 0.1	2.1 ± 0.1	1.6 ± 0.1	1.4 ± 0.1	1.1 ± 0.1
pH	7.6 ± 0.3	7.6 ± 0.4	7.8 ± 0.3	7.8 ± 0.2	7.8 ± 0.4	7.5 ± 0.3	7.5 ± 0.2	7.4 ± 0.1
EC (µS)	0.26 ± 0.01	0.29 ± 0.02	0.27 ± 0.01	0.22 ± 0.01	0.27 ± 0.01	0.19 ± 0.01	0.23 ± 0.01	0.31 ± 0.01
OM (%)	1.5 ± 0.01	3.7 ± 0.03	2.9 ± 0.01	1.5 ± 0.03	1.4 ± 0.02	1.6 ± 0.03	1.4 ± 0.002	1.1 ± 0.01
N (%)	0.18 ± 0.01	0.35 ± 0.03	0.27 ± 0.02	0.23 ± 0.01	0.19 ± 0.01	0.25 ± 0.02	0.14 ± 0.02	0.11 ± 0.01
Moisture (%)	3.2 ± 0.02	24.2 ± 1.2	11.3 ± 0.1	16.7 ± 2.3	8.5 ± 0.9	12.3 ± 1.1	7.5 ± 0.8	5.6 ± 1.2

Values are means of 5 replicates ± standard deviation.

### Floristic composition

**Figure 2:**
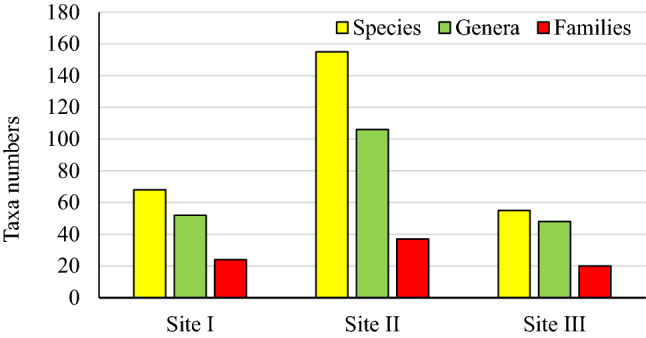
Taxa number recorded in the three sites.

**Figure 3:**
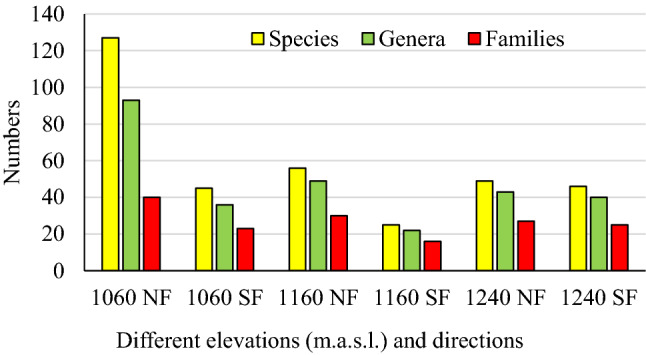
Effect of elevation and slope aspect on taxa numbers in site (II). SF, south face; NF, north face.

The study area comprised 191 species belonging to 131 genera from 43 different families (Supplementary Information: Appendix 2). The current results recorded *Argyrolobium rarum* Dümmer as a new species of the Saudi Arabia flora. The largest family was Gramineae with 20 genera and 24 species, followed by Leguminosae (Fabaceae) and Compositae with 15 species for each. Malvaceae and Euphorbiaceae accounted for ten species each. The three families, Gramineae, Leguminosae, and Compositae contributed to 29% of the total plant species in the current study, whereas 14 families were represented by one species each. *Solanum* was the largest genus with seven species, while each of *Acacia* and *Pulicaria* was represented by six species, followed by *Commicarpos* and *Euphorbia,* each represented by four species. Figure 2 showed that the three sites differed in their taxa numbers, with the middle site exhibiting the highest numbers of species, genera, and families. The fewer taxa numbers were detected at the highest elevation (Site III). The effects of slope aspect and elevation on taxa distribution are presented in Fig. 3. There was no clear pattern of increase or decrease the taxa numbers by increasing the elevation on the south facing side. In contrast to the north facing side, the higher the elevation, the higher the taxa number. The results showed significant differences between the north face and south face directions. The numbers of species, genera, and families on the north facing slopes were higher than those in the south facing ones, which were more obvious at an elevation of 1060 m.a.s.l (Fig. 3). *Aizoon canariense, Achyranthes aspera, Aerva javanica, Calotropis procera,* and *Abutilon bidentatum* were recorded at all three sites.

**Figure 4:**
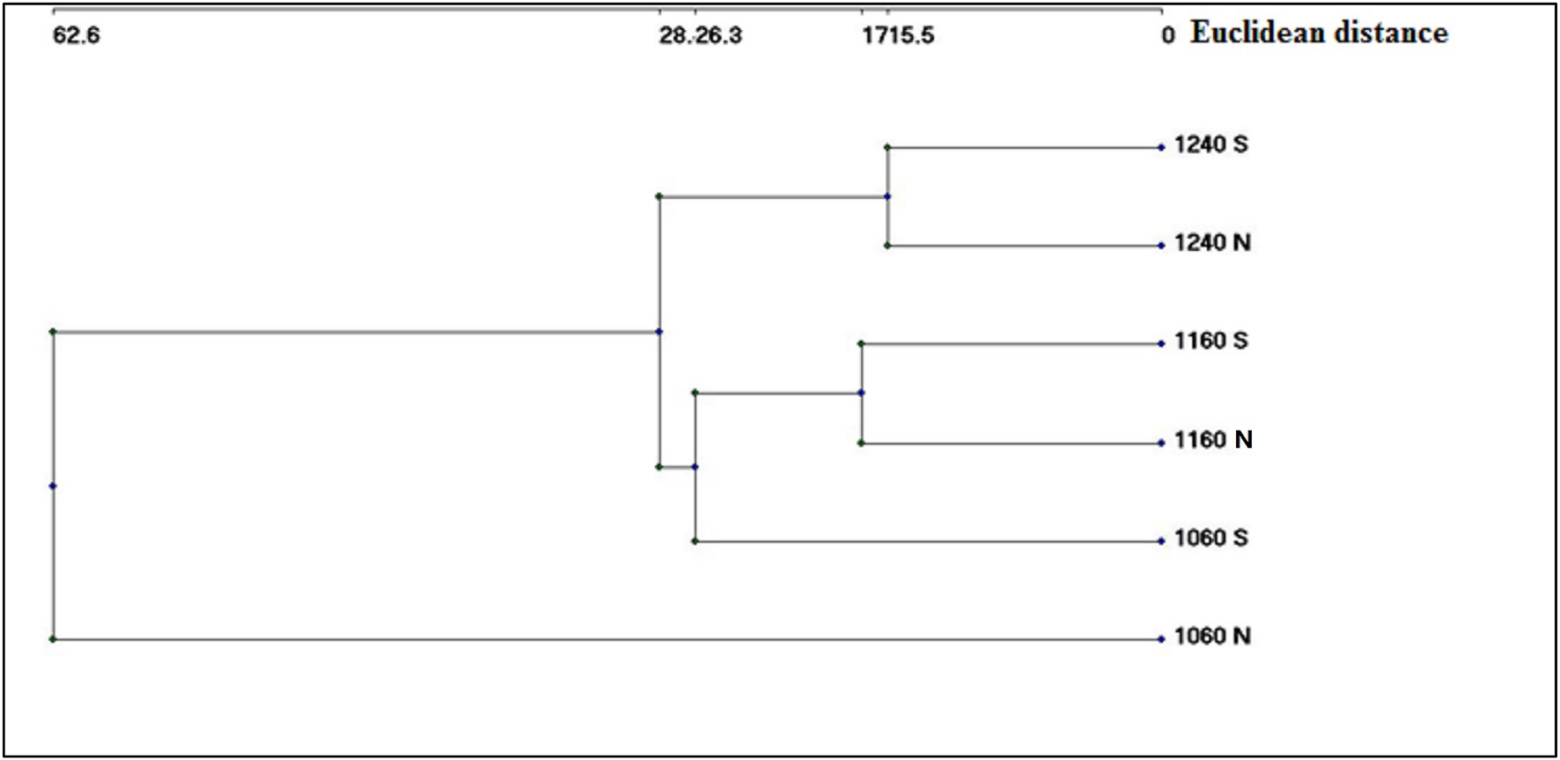
Hierarchical classification of the different belts, in site II, based on their floristic structure (incidence data), by using Ward’s method and Euclidean distances as measurement of Linkage Distance. Elevation value = m.a.sl., N, north facing; S, south facing.

Within site II, *Caralluma retrospiciens*, *Cenchrus pennisetiformis*, *Ocimum forsskaolii*, *Acacia ehrenbergiana*, *Acacia hamulosa*, *Sidda alba*, *Triumfetta flavescens*, *Coccolus pendulus* and *Forsskaolea tenacissima* were recorded in all elevation belts on both north and south facing sides. The Ward classification of different elevation belts on both sides resulted in a dendrogram (Fig. 4).

Figure 4 indicates the effect of both the elevation and the direction on floristic composition, where all belts were divided into two main groups: one represented by 1060 m elevation on the north facing side, and one comprising the remaining belts. The latter is subdivided into 1240 m north and south facing sides, and one with all other categories. The Jaccard similarities showed the one-height belts on both sides (north facing and south facing) were greater than those of different heights in the same direction. Besides, the higher the height, the greater the similarity (Table 3).

**Table 3:** JACCARD similarities between different elevation belts within site II.

	1240 SF	1240 NF	1160 SF	1160 NF	1060 SF	1060 NF
1240 SF	1					
1240 NF	0.5079	1				
1160 SF	0.3846	0.2931	1			
1160 NF	0.3247	0.3636	0.4138	1		
1060 SF	0.3188	0.2703	0.2909	0.2785	1	
1060 NF	0.3106	0.3037	0.186	0.3456	0.3438	1

SF, south facing; NF, north facing.

### Dominant species

*Acacia tortilis* was the dominant species in the lower site (Site I), with *A. hamulosa* as a codominant, while *Ficus palmata* exhibited the dominant species in the upper site (Site III). In site II, *Acacia ehrenbergiana* was the dominant species on both the north and south facing at 1160 m.a.s.l., while *Cenchrus pennisetiformis* and *Triumfetta flavescens* were the codominant species, respectively. *A. ehrenbergiana* also was the dominant species at an elevation of 1240 m.a.s.l. on the north facing side. At an elevation of 1060 m.a.s.l., *Grewia tenax* recorded the dominant species on the north facing slope with *A. hamulosa* as a codominant, while *A. etbaica* was the dominant species on the south facing slope with *c. myrrha* as a codominant*.*

### Life forms

**Figure 5:**
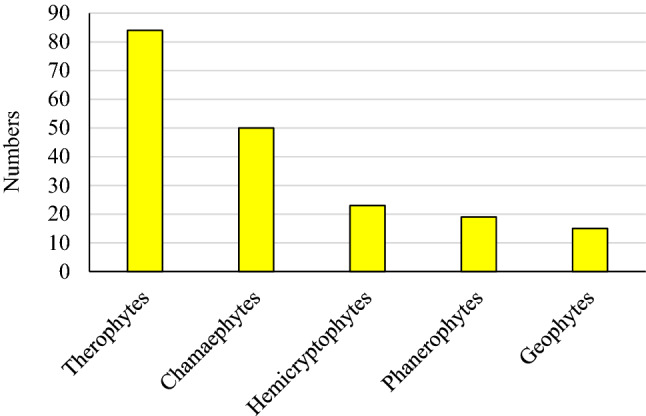
Total life form numbers recorded in all the study areas.

Five life forms were recorded among the total species found in the three study sites. Therophytes exhibited the highest species with 44%, followed by chamaephytes with 26%. Hemicryptophytes and phanerophytes were represented by 12% and 10%, respectively, whereas the lowest life form, 7.8%, was geophytes with 14 species (Table 4, Fig. 5). Life form proportions showed remarkable variations in the studied three sites. Site (III), with the highest elevation, included the highest percentage of therophytes and chaemophytes and the lowest proportions of the other life forms. Phanerophytes in Site II exhibited a higher proportion on the south facing slope than on the north-facing slope at all elevations. The percentages of both chaemophytes and hemicryptophytes recorded remarkable differences between Sit I (750 m a.s.l) and Site III (1830 m.a.s.l). In the upper Site, chaemophytes increased by about 10% and heimicryptophtes decreased by about 58% from the lower site. While geophytes percentages showed a slightly stable ratio along the altitudinal gradient at both aspects.

**Table 4:** Life form percentage (Actual counts between brackets) in the three sites of the studied area, NF, north facing; SF, south facing.

Life forms	Site 1 (68)	Site II (155)						Site 3 (55)
		1060 m.a.s.l		1160 m.a.s.l		1240 m.a.s.l		
		NF (128)	SF (26)	NF (59)	SF (45)	NF (61)	SF (46)	
Chamaephytes	28.0	31.2	34.6	37.2	35.5	39.3	36.9	31.0
Geophytes	4.0	6.2	3.8	3.3	6.6	6.5	6.5	5.0
Hemicryptophytes	12.0	12.5	7.6	10.1	13.3	9.8	8.6	5.0
Phanerophytes	16.0	12.5	19.2	11.8	20.0	13.1	19.5	10.0
Therophytes	40.0	37.5	34.6	37.2	24.4	14.7	6.5	49.0

### Chorological affinities

The chorological composition is summarized in Table 5. Generally, monoregional elements exhibited 26% of the recorded species, while biregional elements recorded 54%. Pluriregional species were represented by 7.8%. The Pantropic elements showed the highest numbers, 9.4%, within the monoregional elements; followed by Saharo-Sindian and Palaeotropical elements, each represented by 4%. The bioregional Saharo-Sindian and Sudano-Zambesian groups were the most represented in the studied area (41%). Cosmopolitan taxa accounted for 8% of the total species, whereas the endemic species, *Kickxia pseudoscoparia*, was recorded in the study area. The chorological affinities shown were variable in their distribution in the studied area. The Pluriregional elements in Site (III) were more than those recorded in Site (I) by 145 folds, with the highest percentage recorded on the north-facing slope at 1060 m a.s.l. In contrast, the Sudano-Zambesian elements have completely disappeared from Site III, Table 6. The bioregional Saharo-Sindian/Sudano-Zambesian, which recorded the highest chorotype in the studied area, increased facing aside from the north facing side in all elevations, an increase ranging from between 13.7 and 35%. Palaeotropical elements decreased by increasing elevation (Table 6) and their proportions were higher on the north facing side than those on the south facing site. In contrast, the bioregional Mediterranean/Irano-Turanian increased at a higher elevation. A slight change was recorded for the Saharo-Sindian elements between Site I and Site III, while it was recorded with higher proportions on the second site, on all elevations for both north and south-facing sides.

**Table 5:** Chorological type’s numbers recorded in the all studied area.

Chorotype	Species	
	Number	%
Saharo-Sindian	16	8.38
Pantropic	17	8.90
Sudano-Zambesian	3	1.57
Mediterranean	1	0.52
Plaeotropical	13	6.81
Saharo-Sindian + Sudano-Zambesian	79	41.36
Saharo-Sindian + Irano-Turanian	6	3.14
Saharo-Sindiani + Mediterranean	7	3.66
Mediterranean + Irano-Turanian	5	2.62
Pantropic + Plaeotropical	7	3.66
Pluriregional	15	7.85
Cosmopolitan	10	5.24
Endemic	1	0.52
Others	11	5.76

**Table 6:** Chorological type’s percentage (actual counts between brackets) recorded in the three different sites, NF, north facing; SF, south facing.

	Site I (68)	Site II (155)						Site III (55)
		1060 m.a.s.l		1160 m.a.s.l		1240 m.a.s.l		
		NF (128)	SF (26)	NF (59)	SF (45)	NF (61)	SF (46)	
Cosmopolitan	6.0	5.0	4.0	7.0	4.0	3.0	2.0	3.6
Plaeotropical	10.0	5.0	4.0	7.0	2.0	0.0	4.0	3.6
Pantropic	6.0	7.0	0.0	8.0	2.0	0.0	0.0	5.5
Sudano-Zambesian	3.0	0.0	4.0	3.0	2.0	3.0	2.0	0.0
Saharo-Sindian	7.0	10.0	19.0	15.0	11.0	16.0	11.0	7.3
Saharo-Sindian + Sudano-Zambesian	44.0	45.0	61.0	50.0	59.0	58.0	66.0	43.6
Saharo-Sindian + Irano-Turanian	3.0	2.0	0.0	0.0	0.0	0.0	2.0	5.5
Mediterranean + Irano-Turanian	4.0	4.0	4.0	2.0	4.0	0.0	0.0	9.1
Endemic	0.0	1.0	0.0	0.0	0.0	2.0	0.0	1.8
Mediterranean	0.0	1.0	0.0	0.0	0.0	0.0	0.0	0.0
Pluriregional	0.1	20.0	4.0	8.0	16.0	18.0	13.0	14.5

## Discussion

The study confirmed that both slope aspect and elevation affect the distribution of plant species, with elevation having the greater impact. In this study, 35% of the recorded families were represented by only one species per family, a prevalent characteristic of desert flora, and considered a sign of plant adaptability to xeric conditions^6^. A large number of families recorded with only one species indicated that only a few species of the many plant families adapt and survive in harsh environments. The species to genera ratio in the current study (1.46) was close to those reported in previous studies of other regions in the Kingdom of Saudi Arabia^6^. According to Magurran^49^ and Pielou^50^, taxonomic diversity is greater in an environment where species are distributed across several genera than in one where the majority of species belong to the same genus. The species per genus ratio is higher than that recorded in the Al-Shafa Highland region^5^ (1.3), and it is less than that reported in the entire Saudi Arabia^6^, 2.6, and lower than the estimated ratios in Khulais^4^. Consequently, the taxonomic diversity of the study area is less diverse than in the Al-Shafa heights and greater than in the Khulais region and all of Saudi Arabia. This result indicates that the taxonomic diversity is increasing with increasing height in the west of the Kingdom of Saudi Arabia.

The dominance of Gramineae, Leguminosae, and Compositae and the patterns of the most different families were consistent with previous studies of other regions in Saudi Arabia, such as Khulais^4^, Al-shafa highland^5^, the Asir Mountains^51^, and Hail^52^. Moreover, the current results are like those of neighboring countries, which include Egypt^53^ and Sudan, in the Marra Mountains^43^. Only one endemic species was recorded inside the studied area, *Kickxia pseudoscoparia*. This is in agreement with Al-Nafie^6^, who stated that endemism in Saudi Arabia is insignificant in comparison to some of the adjoining nations, such as Yemen and Oman. The highest taxa numbers in the middle part of the study area (Site II) may be attributed to the minor impact of human disturbances in this section. This site is far from the road, while the first and third sites are close to it (Fig. 1). The observed widespread species in all elevation belts in the north facing and south facing sides could be attributed to their wide ecological amplitude. The contents of total nitrogen, organic matter, and moisture in the soils of the north facing side at 1060 m a.s.l have made suitable conditions for many plants, resulting in a large increase in all taxa numbers.

It has been recorded that in areas where annual precipitation is less than 600 mm, moisture becomes an important factor in the composition, intensity, and structure of plant communities^33^. Besides, it was argued that soil moisture was the main environmental issue governing plant species composition and richness^54^.

Small differences in soil analysis were found among the studied belts, except at 1060 m a.s.l on the north facing slope. This result is not in line with Kutiel and Lavee^33^, who stated that considerable variations in soil properties between the opposing directions can be predicted in arid zones (< 400 mm of annual precipitation) due to low rainfall and high potential evaporation. Our finding is in line with Burke's^55^ study of Namibia's arid Nama Karoo inselbergs.

The higher numbers of recorded taxa in all the north facing belts than those in the south facing belts can be explained because the south facing slopes receive more sunlight and become warmer and xeric, helping drought-resistant flora, and are less conducive to tree growth, while the north facing slopes preserve moisture and are humid, supporting moisture-loving plants^56^. It has been reported that insolation affects greatly the composition of plant species, particularly in arid and semiarid regions at low latitudes, through its impact on the water balance^57^. In addition, high evapotranspiration on the south facing slopes may cause drought stress during the growing season. *Acacia tortilis*, which was the dominant species at the lowest site, was recorded as well adapted to disturbances such as drought, fire, browsing, and pollarding^58^. *Ficus palmata*, the dominant species on the upper site is distributed from Egypt to Central Asia and is typical of the cold desert mountains of this area. Sometimes *Ficus palmata* subsp. *virgata* is also confused with its closely allied species, *F*. *carica*, a native of the Mediterranean region to Afghanistan^59^.

The high percentage of therophytes (44%) in the study area agrees with previous studies in other parts of Saudi Arabia^4,6,40,41,52,60–62^. A similar result was published for the northern part of the Eastern Desert of Egypt^63^. Sharma and Rajpal^64^ also reported that in semi-deserts, therophytes typically contribute about 30% to 55% of all life forms.

Previous publications indicate that topography affects the life forms of desert plants^39,44,51,65–69^. The current results are in full agreement with Cain and Castro^70^ and Pavón et al.^13^, who stated that therophytes and chamaephytes increased in dominance and/or abundance with increasing elevation. In addition, the therophytes percentage in the current study mediates the proportions of the Khulais, with a lower elevation^4^, and the Al-shafa highland regions, with a higher elevation^5^. Chamaephytes distribution in the current study contrasts with the observations of Dickoré and Nüsser^71^, who reported that chamaephytes decrease with increasing elevation. This discrepancy between the current study and the study of Dickoré and Nüsser^71^ could be attributed to the large difference in elevation (1200–1400 m a.s.l.) to 3000–3400 m a.s.l. Phanerophytes distribution in our results agrees with the finding of Hoffmann and Hoffmann^72^, who reported that phanerophytes decrease in a temperate area and are very common in tropical regions. The percent of Phanerophytes recorded in this study agrees with White and Leonard^73^ statement that “the southern and southwestern Arabian Peninsula is very poor in trees”.

Numerous early studies express that the flora and vegetation of Saudi Arabia have included elements from two main phytogeographical regions that cover much of North Africa and East Asia, the Sudano-Zambezian region and the Saharo-Arabian-Sindian region^71–75^. The studied region belongs to the Nubo-Sindian Province, which is a part of the Sudanian region^44^. Wickens^43^ recorded that the current examined area is principally impacted by the Saharo-Sindian element, whereas White and Leonard^73^ stated that the Sudano-Zambezian region stretches out into southern and western Arabia. These influences clarify why the highest percentages for monoregional, bioregional, and pluriegional species have been recorded for the Saharo-Sindian elements, followed by those of the Sudano-Zambesian. In addition, the White and Leonard^73^ statement explains that the bioregional Saharo-Sindian and Sudano-Zambesian groups were the most represented in the studied area. Alsherif et al.^4^ recorded that Saharo-Arabian and Sudanian elements exhibited the highest chronological elements in the Khulais region, a region away from the current location by 140 km in the northwest direction. Al-sherif and Fadl^5^ recorded that Saharo-Sindian had the most bioregional groups in the Alshafa highlands (1700–2300 m a.s.l). The biregional elements Saharo-Sindian and Sudano-Zambesian biregional elements were more frequent on the southern rather than northern facing slopes because the southern slopes experienced warmer and drier conditions than the northern slopes. Sudano-Zambesian elements were most frequent at lower elevations and decreased in frequency with rising elevation, possibly because these elements rely on tropical climatic conditions6. In contrast, a high percentage of Saharo-Sindian and Irano-Turanian elements were found at high elevations^6^. On the contrary, a high percent of Saharo-Sindian and Irano-Turanian elements were found at high elevations.

## Conclusions

In the current study of a valley that cuts through the Sarawat Mountains, we identified a range of Palaearctic flora co-existing with Afrotropical plants. It is the first study of the influence of slope direction in the distribution of plant species in the Sarawat Mountains, and it sheds light on the study area in terms of its floristic significance. The study found that both slope aspects and elevation have an impact on plant distribution, with the influence of elevation being the most important. Since south facing slopes receive more sunlight and become warmer and xeric, the higher numbers of the reported taxa in all north facing belts than in the south facing belts can be explained. The report of the new addition to the flora of Saudi Arabia (*Argyrolobium rarum*) showed that the Sarawat mountains need careful botanical exploration. We recommend using LiDAR data as part of future research.

## Supplementary Information

Supplementary Information 1.

Supplementary Information 2.

## References

[1] Cunningham SC, Mac Nally R, Baker PJ, Cavagnaro TR, Beringer J, Thomson JR, Thompson RM (2015). Balancing the environmental benefits of reforestation in agricultural regions. Perspect. Plant Ecol. Evol. Syst..

[2] Pearse IS, Hipp AL (2009). Phylogenetic and trait similarity to a native speciespredict herbivory on non-native oaks. Proc. Natl. Acad. Sci. U. S. A..

[3] Abdel Khalik K, El-Sheikh M, El-Aidarous A (2013). Floristic diversity and vegetation analysisof wadi Al Noman, Holy Mecca, Saudi Arabia. Turk. J. Bot..

[4] Al-Sherif EA, Ayesh AM, Rawi SM (2013). Floristic composition, life form and chorology of plant life at Khulais region western Saudi Arabia. Pak. J. Bot..

[5] Al-Sherif EA, Fadl MA (2016). Floristic study of the Al-Shafa Highlands in Taif, western Saudi Arabia. Flora.

[6] Al-Nafie AH (2008). Phytogeography of Saudi Arabia. Saudi J. Biol. Sci..

[7] Mossa JS, Al-Yahya MA, Al-Meshal IA (1987). Medicinal Plants of Saudi Arabia.

[8] Körner C (2000). Why are there global gradients in species richness? Mountains might hold the answer. Trends Ecol. Evol..

[9] Cano-Ortiz A, Musarella CM, PiNar Fuentes JC, Gomes CJP, Cano E (2016). Distribution patterns of endemic flora to define hotspots on Hispaniola. Syst. Biodiv..

[10] Hedberg, O. The flora of Ethiopia: a progress report. in *Research in Ethiopia Flora* (ed. Hedberg, I.). Symb. Bot. Ups. **26**, 17–18 (1986).

[11] Cowling RM, Esler KJ, Midgley GF, Honing MA (1994). Plant functional diversity, species diversity and climate in arid and semi-arid southern Africa. J. Arid Environ..

[12] Montana C, Valientebanuet A (1998). Floristic and life-form diversity along an altitudinal gradient in an intertropical semiarid Mexican region. Southwest. Nat..

[13] Pavón NP, Hernández-Trejo H, Rico-Gray V (2000). Distribution of plant lifeforms along an altitudinal gradient in the semi-arid valley of Zapotitlón, Mexico. J. Veg. Sci..

[14] Raunkiaer C (1910). Statistik der Lebensformen als Grundlage für die biologische Pflanzengeographie. Beih. Bot. Centralbl..

[15] Sarmiento G, Monasterio M, Bourlièrre F (1983). Life form and phenology. Tropical Savannas.

[16] Meher-Homji VM (1981). Environmental implications of life-form spectra from India. J. Econ. Tax. Bot..

[17] Campbell BM, Werger MJA (1988). Plant form in mountains of the Cape, South Africa. J. Ecol..

[18] Komárková V, McKendrick JD, Werger MJA, van der Aart PJM, During HJ, Verhoeven JTA (1988). Patterns in vascular plant growth forms in arctic communities and environment at Atkasook, Alaska. Plant Form and Vegetation Structure.

[19] Cody ML (1989). Growth-form diversity and community structure in desert plants. J. Arid Environ..

[20] Danin A, Orshan G (1990). The distribution of Raunkiaer life forms in Israel in relation to the environment. J. Veg. Sci..

[21] Osman AK, Al-Ghamdi F, Bawadekji A (2014). Floristic diversity and vegetation analysis of Wadi Arar: a typical desert Wadi of the Northern Border region of Saudi Arabia. Saud. J. Biol. Sci..

[22] Grime JP (1979). Plant Strategies and Vegetation Processes.

[23] Palmer MW (1992). The coexistence of species in fractal landscapes. Am. Nat..

[24] Huston M, DeAngelis DL (1994). Competition and coexistence: the effects of resource transport and supply rates. Am. Nat..

[25] Szaro RC (1989). Riparian forest and scrubland communities of Arizona and New Mexico. Desert Plants.

[26] DeBano LF, Schimdt LJ (1990). Potential for enhancing riparian habitat in the Southwestern United States with watershed practices. For. Ecol. Manag..

[27] Lieberman D, Lieberman M, Peralta R, Hartshorn GS (1996). Tropical forest structure and composition on a large-scale altitudinal gradient in Costa Rica. J. Ecol..

[28] Zimmerman JC, DeWald LE, Rowlands PG (1999). Vegetation diversity in an interconnected ephemeral riparian system of north-central Arizona, USA. Biol. Conserv..

[29] Brown J (2001). Mammals on mountainsides: elevational patterns of diversity. Glob. Ecol. Biogeogr..

[30] Lomolino MV (2001). Elevation gradients of species-density: historical and prospective views. Glob. Ecol. Biogeogr..

[31] Ahmed MJ, Murtaza G, Shaheen H, Habib T (2020). Distribution pattern and associated flora of *Jurinea dolomiaea* in the western Himalayan highlands of Kashmir: an indicator endemic plant of alpine phytodiversity. Ecol. Ind..

[32] Bhat JA, Kumarc M, Pala NA, Shaha S, Dayala S, Gunathilake C, Negic AK (2020). Influence of altitude on the distribution pattern of flora in a protected area of Western Himalaya. Acta Ecol. Sin..

[33] Kutiel P, Lavee H (1999). Effect of slope aspect on soil and vegetation properties along an aridity transect. Isr. J. Plant Sci..

[34] Cantlon J (1953). Vegetation and microclimates of north and south slopes of Cushetunk mountain. New Jersey. Ecol. Monogr..

[35] Vetaas OR (1992). Gradients in field-layer vegetation on an arid misty mountain plateau in the Sudan. J. Veg. Sci..

[36] Kirkpatrick J, Fensham R, Nunez M, Bowman D (1998). Vegetation-radiation relation in the wet-dry tropics: granite hills in northern Australia. Vegetatio.

[37] Ady J (1995). The Taif escarpment, Saudi Arabia: a study for nature conservation and recreational development. Mt. Res. Dev..

[38] Almazroui M, Nazrul Islam M, Athar H, Jones PD, Rahman MA (2012). Recent climate change in the Arabian Peninsula: annual rainfall and temperature analysis of Saudi Arabia for 1978–2009. Int. J. Climatol..

[39] Migahid AM (1996). Flora of Saudi Arabia.

[40] Collenette S (1999). Wild Flowers of Saudi Arabia.

[41] Chaudhary S (2001). Flora of the Kingdom of Saudi Arabia.

[42] Raunkiaer C (1934). Life Forms of Plants and Statistical Plant Geography (Collected Paper Translated into English).

[43] Wickens GE (1976). The Flora of Jebel Morra (Sudan Republic) and Its Geographical Affinities. Kew Bulletin Additional Series V.

[44] Zohary M (1973). Geobotanical Foundations of the Middle East.

[45] Broadbent FE, Black CA (1965). Organic matter. Methods of Soil Analysis Part 1.

[46] Bremmer JM, Black CA (1965). Total nitrogen. Methods of Soil Analysis Part 1.

[47] Ward JH (1963). Hierarchical grouping to optimize an objective function. Am. Stat. Assoc. J..

[48] Castro SA, Jaksic FM (2008). Patterns of turnover and floristic similarity show a non random distribution of naturalized flora in Chile. South America. Rev. Hist. Nat..

[49] Magurran AE (1988). Ecological Diversity and Its Measurements.

[50] Pielou EC (1975). Ecological Diversity.

[51] Hosni HA, Hegazy AK (1996). Contribution to the flora of Asir, Saudi Arabia. Candollea.

[52] Al-Turki TA, Al-Olayan HA (2003). Contribution to the flora of Saudi Arabia: hail region. Saud. J. Biol. Sci..

[53] Abd El-Ghani MM, Abdel-Khalik KN (2006). Floristic diversity and phytogeography of the gebel Elba national park South-East Egypt. Turk. J. Bot..

[54] Panthi MP, Chaudhary RP, Vetaas OR (2007). Plant species richness and composition in a trans Himalayan inner valley of mananging district, Central Nepal. Himal. J. Sci..

[55] Burke A (2002). Properties of soil pockets on arid Nama karoo inselbergsethe effect of geology and derived landforms. J. Arid Environ..

[56] Måren IE, Karki S, Prajapati C, Yadav RK, Shrestha BB (2015). Facing north or south: does slope aspect impact forest standcharacteristics and soil properties in a semiarid trans-Himalayanvalley?. J. Arid Environ..

[57] Boyko H (1947). On the role of plants as quantitative climate indicators and the geoecological law of distributions. J. Ecol..

[58] Andersen GL, Krzywinski K (2007). Longevity and growth of *Acacia tortilis*; insights from 14C content and anatomy of wood. BMC Ecol..

[59] Tiwari N, Srivastava N, Sharma V (2014). Comparative analysis of total phenolic content and antioxidant activity of in vivo and in vitro grown plant parts of *Carica papaya* L. Ind. J. Plant Physiol..

[60] Daur I (2012). Plant flora in the rangeland of Western Saudi Arabia. Pak. J. Bot..

[61] El-Demerdash MA, Hegazy AK, Zilay AM (1994). Distribution of plant communities in Tihamah coastal plains of Jazan region, Saudi Arabia. Vegetatio.

[62] El-Ghanim WM, Hassan LM, Galal TM, Badr A (2010). Floristic composition and vegetation analysis in Hail region north of Central Saudi Arabia. Saudi J. Biol. Sci..

[63] Abd El-Ghani MM (1998). Environmental correlates of species distribution in arid desert ecosystems of eastern Egypt. J. Arid Environ..

[64] Sharma M, Rajpal K (1991). Life-forms and biological spectrum of the flora of the Punjab state, India. Bull. Bot. Surv. India.

[65] Hegazy AK, El-Demerdash MA, Hosni HA (1998). Vegetation, species diversity and floristic relations along an altitudinal gradient in South-West Saudi Arabia. J. Arid Environ..

[66] Kassas M, Girgis WA (1964). Habitats and plant communities in the Egyptian deserts. V. The limestone plateau. J. Ecol..

[67] Orshan G, Evenari M, Meir NI, Good DW (1986). The desert of the middle east. Ecosystems of the World, 12B, Hot Desert and Arid Shrublands.

[68] Shaltout KH, Sheded MG, Salem AM (2010). Vegetation spatial heterogeneity in a hyper arid biosphere reserve area in North Africa. Act. Bot. Croat..

[69] Stewart L (2016). The regional species richness and genetic diversity of Arctic vegetation reflect both past glaciations and current climate. Ecol. Biogeogr..

[70] Cain SA, Castro MO (1959). Manual of Vegetation Analysis.

[71] Dickoré WB, Nüsser M (2000). Flora of Nanga Parbat (NW Himalaya, Pakistan): an annotated inventory of vascular plants with remarks on vegetation dynamics. Englera.

[72] Hoffmann AJ, Hoffmann AE (1982). Altitudinal ranges of phanerophytes and chamaephytes in central Chile. Vegetatio.

[73] White F, Leonard J (1991). Phytogeographical links between Africa and Southwest Asia. Flora Veg. Mundi..

[74] König P (1988). Phytogeography of South-Western Saudi Arabia (Asir, Tihama). Erde.

[75] White F (1983). The vegetation of Africa: A descriptive memoir to accompany the UNSECO, AETFAT, UNSO vegetation map of Africa.

