# Supplementary material for: Impact of elevation and slope aspect on floristic composition in wadi Elkor, Sarawat Mountain, Saudi Arabia
Source: Sci Rep. 2021 Aug 9;11:16160. doi: 10.1038/s41598-021-95450-4 (PMC8352965; doi:10.1038/s41598-021-95450-4)
Supplement: Supplementary file 1 — Supplementary Information 1. [file 41598_2021_95450_MOESM1_ESM.docx]

**Appendix 1:  ID number for the voucher specimen, Species are arranged alphabetically.**

| **Specimen name** | **ID number for the voucher specimen** |
| --- | --- |
| *Bidens biternata* (Lour) Merr. & Sherrf | Wadi ELKor, 2019, 10027 (TUH) Fadl, M.A. & Alsherif, E.A. |
| *Boerhavia diffusa* L. | Wadi ELKor, 2019, 10076 (TUH) Fadl, M.A. & Alsherif, E.A. |
| *Brachiaria leersioides* (Hochst.) Stapf | Wadi ELKor, 2019, 10046 (TUH) Fadl, M.A. & Alsherif, E.A. |
| *Bromus diandrus* Roth | Wadi ELKor, 2019, 10047 (TUH) Fadl, M.A. & Alsherif, E.A. |
| *Cadaba farinose* Forssk. | Wadi ELKor, 2019, 10024 (TUH) Fadl, M.A. & Alsherif, E.A. |
| *Calotropis procera* (Ait.) Ait. f. | Wadi ELKor, 2020, 10205 (TUH) Fadl, M.A. & Alsherif, E.A. |
| *Capparis spinosa* L. | Wadi ELKor, 2020, 10235 (TUH) Fadl, M.A. & Alsherif, E.A. |
| *Caralluma retrospiciens* (Ehrenb.) N.E. Br. | Wadi ELKor, 2020, 10206 (TUH) Fadl, M.A. & Alsherif, E.A. |
| *Cassia italic* | Wadi ELKor, 2020, 10299 (TUH) Fadl, M.A. & Alsherif, E.A. |
| *Cenchrus ciliaris* L. | Wadi ELKor, 2019, 10048 (TUH) Fadl, M.A. & Alsherif, E.A. |
| *Cenchrus longisetus ****M.C.Johnst. | Wadi ELKor, 2019, 10050 (TUH) Fadl, M.A. & Alsherif, E.A. |
| *Cenchrus pennisetiformis* Hochst. & Stedudel ex Steudel | Wadi ELKor, 2019, 10049 (TUH) Fadl, M.A. & Alsherif, E.A. |
| *Cenchrus setigerus* Vahl | Wadi ELKor, 2020, 10278 (TUH) Fadl, M.A. & Alsherif, E.A. |
| *Chenopodium carinatum* R.Br. | Wadi ELKor, 2020, 10244 (TUH) Fadl, M.A. & Alsherif, E.A. |
| *Chenopodium murale* L. | Wadi ELKor, 2020, 10245 (TUH) Fadl, M.A. & Alsherif, E.A. |
| *Chenopodium opulifolium* Schrader ex Koch & Ziz | Wadi ELKor, 2020, 10246 (TUH) Fadl, M.A. & Alsherif, E.A. |
| *Chrozophora oblongifolia* (Del.) A. Juss. ex Spreng | Wadi ELKor, 2019, 10034 (TUH) Fadl, M.A. & Alsherif, E.A. |
| *Chrysopogon plumulosus* Hochst. | Wadi ELKor, 2020, 10279 (TUH) Fadl, M.A. & Alsherif, E.A. |
| *Citrullus colocynthis* (L.) Schrader | Wadi ELKor, 2020, 10272 (TUH) Fadl, M.A. & Alsherif, E.A. |
| *Citrullus lanatus* (Thunb.) Matsumara & Nakai | Wadi ELKor, 2020, 10273 (TUH) Fadl, M.A. & Alsherif, E.A. |
| *Cleome gynandra* L. | Wadi ELKor, 2020, 10250(TUH) Fadl, M.A. & Alsherif, E.A. |
| *Cleome hanburyana* Penz. | Wadi ELKor, 2020, 10251 (TUH) Fadl, M.A. & Alsherif, E.A. |
| *Cleome ramosissima* Webb. | Wadi ELKor, 2020, 10252 (TUH) Fadl, M.A. & Alsherif, E.A. |
| *Cleome scaposa* DC. | Wadi ELKor, 2019, 10025 (TUH) Fadl, M.A. & Alsherif, E.A. |
| *Coccinea grandis* (L.) Voigt. | Wadi ELKor, 2020, 10271 (TUH) Fadl, M.A. & Alsherif, E.A. |
| *Coccolus pendulus* (J.R. & G. Forster) Diels | Wadi ELKor, 2020, 10308 (TUH) Fadl, M.A. & Alsherif, E.A. |
| *Cometes abyssinica* R.Br. | Wadi ELKor, 2020, 10240 (TUH) Fadl, M.A. & Alsherif, E.A. |
| *Commelina benghalensis* L. | Wadi ELKor, 2019, 10026 (TUH) Fadl, M.A. & Alsherif, E.A. |
| *Commicarpos ambiguus* Meikle | Wadi ELKor, 2019, 10077 (TUH) Fadl, M.A. & Alsherif, E.A. |
| *Commicarpos grandiflorus* (A. Rich.) Standley | Wadi ELKor, 2019, 10078 (TUH) Fadl, M.A. & Alsherif, E.A. |
| *Commicarpos helenae* (Roem. & Schultes) Meikle | Wadi ELKor, 2019, 10079 (TUH) Fadl, M.A. & Alsherif, E.A. |
| *Commicarpos plumbagineus* (Cav.) Standley | Wadi ELKor, 2019, 10080 (TUH) Fadl, M.A. & Alsherif, E.A. |
| *Commiphora habissinica* (O. Berg.) Engl. | Wadi ELKor, 2019, 10022 (TUH) Fadl, M.A. & Alsherif, E.A. |
| *Commiphora kataf* Engl. | Wadi ELKor, 2019, 10021 (TUH) Fadl, M.A. & Alsherif, E.A. |
| *Commiphora myrrha* Engl. | Wadi ELKor, 2019, 10023 (TUH) Fadl, M.A. & Alsherif, E.A. |
| *Convolvulus arvensis* L. | Wadi ELKor, 2020, 10263 (TUH) Fadl, M.A. & Alsherif, E.A. |
| *Conyza bonariensis* (L.) Cronq. | Wadi ELKor, 2019, 10028 (TUH) Fadl, M.A. & Alsherif, E.A. |
| *Corbichonia decumbens* (Forssk.) Exell | Wadi ELKor, 2020, 10309 (TUH) Fadl, M.A. & Alsherif, E.A. |
| *Crotalaria emarginella* | Wadi ELKor, 2020, 10300 (TUH) Fadl, M.A. & Alsherif, E.A. |
| *Crotalaria microphylla* | Wadi ELKor, 2020, 10301(TUH) Fadl, M.A. & Alsherif, E.A. |
| *Cucumis prophetarum var. prophetarum* L. | Wadi ELKor, 2020, 10274 (TUH) Fadl, M.A. & Alsherif, E.A. |
| *Cynanchum acutum* L. | Wadi ELKor, 2020, 10207 (TUH) Fadl, M.A. & Alsherif, E.A. |
| *Cynodon dactylon* (L.) Pers. | Wadi ELKor, 2020, 10280 (TUH) Fadl, M.A. & Alsherif, E.A. |
| *Datura innoxia* Mill. | Wadi ELKor, 2019, 10101 (TUH) Fadl, M.A. & Alsherif, E.A. |
| *Datura stramonium* L | Wadi ELKor, 2019, 10102 (TUH) Fadl, M.A. & Alsherif, E.A. |
| *Digera muricata* (L.) Mart. | Wadi ELKor, 2020, 10203 (TUH) Fadl, M.A. & Alsherif, E.A. |
| *Digitaria sanguinalis* (L.) Scop. | Wadi ELKor, 2020, 10281 (TUH) Fadl, M.A. & Alsherif, E.A. |
| *Ecbolium viride* Alston | Wadi ELKor, 2020, 10200 (TUH) Fadl, M.A. & Alsherif, E.A. |
| *Echinochloa colona* (L.) Link | Wadi ELKor, 2020, 10282 (TUH) Fadl, M.A. & Alsherif, E.A. |
| *Echinops hystrichoides* Kit-Tan | Wadi ELKor, 2019, 10029 (TUH) Fadl, M.A. & Alsherif, E.A. |
| *Echium longiflorum* Del. | Wadi ELKor, 2019, 100014 (TUH) Fadl, M.A. & Alsherif, E.A. |
| *Eleusine indica ssp. Indica* (L.) Gaertn. | Wadi ELKor, 2020, 10285 (TUH) Fadl, M.A. & Alsherif, E.A. |
| *Ephedra foliate* Boiss. ex C.A. May | Wadi ELKor, 2020, 10276 (TUH) Fadl, M.A. & Alsherif, E.A. |
| *Eragrostis pilosa* (L.) P. Beauv. | Wadi ELKor, 2020, 10283 (TUH) Fadl, M.A. & Alsherif, E.A. |
| *Euphorbia granulata* Forssk. | Wadi ELKor, 2019, 10036 (TUH) Fadl, M.A. & Alsherif, E.A. |
| *Euphorbia arabica* Hochst. & Wteyd | Wadi ELKor, 2019, 10035 (TUH) Fadl, M.A. & Alsherif, E.A. |
| *Euphorbia prostrata* Aiton | Wadi ELKor, 2019, 10037 (TUH) Fadl, M.A. & Alsherif, E.A. |
| *Euphorbia schimperi* Presl. | Wadi ELKor, 2019, 10038 (TUH) Fadl, M.A. & Alsherif, E.A. |
| *Euphorbia serpens* Kunth | Wadi ELKor, 2019, 10039 (TUH) Fadl, M.A. & Alsherif, E.A. |
| *Fagonia indica* Burm.f. | Wadi ELKor, 2019, 10093 (TUH) Fadl, M.A. & Alsherif, E.A. |
| *Fagonia paulayana* Wagner & Verh. | Wadi ELKor, 2019, 10094 (TUH) Fadl, M.A. & Alsherif, E.A. |
| *Farsetia longisiliqua* Decne. | Wadi ELKor, 2020, 10266 (TUH) Fadl, M.A. & Alsherif, E.A. |
| *Ficus cordata ssp. salicifolia* (Vahl) C.C. Berg. | Wadi ELKor, 2019, 10073 (TUH) Fadl, M.A. & Alsherif, E.A. |
| *Ficus palmata* Forssk. | Wadi ELKor, 2019, 10074 (TUH) Fadl, M.A. & Alsherif, E.A. |
| *Ficus sycomorus* | Wadi ELKor, 2019, 10075 (TUH) Fadl, M.A. & Alsherif, E.A. |
| *Fimbristylis turkistanica* (Regel) B. Fedtsch. | Wadi ELKor, 2020, 10275 (TUH) Fadl, M.A. & Alsherif, E.A. |
| *Forsskaolea tenacissima* L. | Wadi ELKor, 2019, 10091 (TUH) Fadl, M.A. & Alsherif, E.A. |
| *Glossonema boveanum* (Decne) Decne | Wadi ELKor, 2020, 10219 (TUH) Fadl, M.A. & Alsherif, E.A. |
| *Grewia erythraea* Schweinf. | Wadi ELKor, 2019, 10068 (TUH) Fadl, M.A. & Alsherif, E.A. |
| *Grewia tenax* (Forssk.) Fiori | Wadi ELKor, 2019, 10069 (TUH) Fadl, M.A. & Alsherif, E.A. |
| *Heliotropium arbainense* Fresen. | Wadi ELKor, 2019, 10015 (TUH) Fadl, M.A. & Alsherif, E.A. |
| *Heliotropium longiflorum* (Hochst. & Steud.) Jaub. & Spach. | Wadi ELKor, 2019, 10016 (TUH) Fadl, M.A. & Alsherif, E.A. |
| *Heliotropium strigosum* Willd. | Wadi ELKor, 2019, 10017 (TUH) Fadl, M.A. & Alsherif, E.A. |
| *Heliotropium subulatumn* (DC.) Vatke | Wadi ELKor, 2019, 10018 (TUH) Fadl, M.A. & Alsherif, E.A. |
| *Hibiscus micranthus* L. | Wadi ELKor, 2019, 10070 (TUH) Fadl, M.A. & Alsherif, E.A. |
| *Indigofera articulata Nov****Gouan | Wadi ELKor, 2020, 10302(TUH) Fadl, M.A. & Alsherif, E.A. |
| *Indigofera hochstetteri* Bak*.* | Wadi ELKor, 2020, 10303 (TUH) Fadl, M.A. & Alsherif, E.A. |
| *Indigofera spinosa* Forssk*.* | Wadi ELKor, 2020, 10304 (TUH) Fadl, M.A. & Alsherif, E.A. |
| *Indigofera trita ssp. subulata* | Wadi ELKor, 2019, 10062 (TUH) Fadl, M.A. & Alsherif, E.A. |
| *Justicia flava* (Vahl) Vahl | Wadi ELKor, 2020, 10201 (TUH) Fadl, M.A. & Alsherif, E.A. |
| *Kickxia pseudoscoparia* V.W. Smith | Wadi ELKor, 2020, 10311 (TUH) Fadl, M.A. & Alsherif, E.A. |
| *Lavandula coronopifolia* Poir. | Wadi ELKor, 2019, 10054 (TUH) Fadl, M.A. & Alsherif, E.A. |
| *Lavandula pubescens* Decne. | Wadi ELKor, 2019, 10055 (TUH) Fadl, M.A. & Alsherif, E.A. |
| *Leptadenia pyrotechnica* (Forssk.) Decne | Wadi ELKor, 2020, 10220 (TUH) Fadl, M.A. & Alsherif, E.A. |
| *Lindenbergia indica var. indica* (L.) Vatke | Wadi ELKor, 2020, 10312 (TUH) Fadl, M.A. & Alsherif, E.A. |
| *Lycium shawii* Roem. & Schult. | Wadi ELKor, 2019, 10103 (TUH) Fadl, M.A. & Alsherif, E.A. |
| *Malva parviflora* L. | Wadi ELKor, 2019, 10071 (TUH) Fadl, M.A. & Alsherif, E.A. |
| *Melhania philippsiae* Baker | Wadi ELKor, 2019, 10072 (TUH) Fadl, M.A. & Alsherif, E.A. |
| *Mentha longifolia ssp. schimperi* (Briq.) Briq. | Wadi ELKor, 2019, 10056 (TUH) Fadl, M.A. & Alsherif, E.A. |
| *Morettia canescens* Boiss. | Wadi ELKor, 2020, 10267 (TUH) Fadl, M.A. & Alsherif, E.A. |
| *Morettia parviflora* Boiss. | Wadi ELKor, 2020, 10269 (TUH) Fadl, M.A. & Alsherif, E.A. |
| *Ochradenus baccatus* Del. | Wadi ELKor, 2019, 10086 (TUH) Fadl, M.A. & Alsherif, E.A. |
| *Ocimum forsskaolii* Benth. | Wadi ELKor, 2019, 10057 (TUH) Fadl, M.A. & Alsherif, E.A. |
| *Osteospermum vaillantii* (Decne.) Norlindh | Wadi ELKor, 2019, 10030 (TUH) Fadl, M.A. & Alsherif, E.A. |
| *Otostegia fruticosa ssp. schimperi* (Benth.) Sebald | Wadi ELKor, 2019, 10058 (TUH) Fadl, M.A. & Alsherif, E.A. |
| *Panicum repens* L. | Wadi ELKor, 2020, 10284 (TUH) Fadl, M.A. & Alsherif, E.A. |
| *Peganum harmala* L. | Wadi ELKor, 2019, 10095 (TUH) Fadl, M.A. & Alsherif, E.A. |
| *Pennisetum orientale* L.C. Rich | Wadi ELKor, 2020, 10286 (TUH) Fadl, M.A. & Alsherif, E.A. |
| *Pergularia daemia* (Forssk.) Chiov. | Wadi ELKor, 2020, 10221 (TUH) Fadl, M.A. & Alsherif, E.A. |
| *Pergularia tomentosa* L. | Wadi ELKor, 2020, 10231 (TUH) Fadl, M.A. & Alsherif, E.A. |
| *Peristrophe paniculata*(Forssk.) Brummit | Wadi ELKor, 2020, 10202 (TUH) Fadl, M.A. & Alsherif, E.A. |
| *Phoenix dactyliferae* | Wadi ELKor, 2019, 10081 (TUH) Fadl, M.A. & Alsherif, E.A. |
| *Phragmites australis* (Cav.) Trin. & Steudel. | Wadi ELKor, 2020, 10287 (TUH) Fadl, M.A. & Alsherif, E.A. |
| *Phyllanthus rotundifolius* | Wadi ELKor, 2019, 10040 (TUH) Fadl, M.A. & Alsherif, E.A. |
| *Phyllanthus tenellus* Roxb. | Wadi ELKor, 2019, 10041 (TUH) Fadl, M.A. & Alsherif, E.A. |
| *Plectranthus tenuiflorus* (Vatke) Agnew | Wadi ELKor, 2019, 10059 (TUH) Fadl, M.A. & Alsherif, E.A. |
| *Pluchea dioscoridis* (L.) DC. | Wadi ELKor, 2019, 10031 (TUH) Fadl, M.A. & Alsherif, E.A. |
| *Portulaca oleraceae* L. | Wadi ELKor, 2019, 10084 (TUH) Fadl, M.A. & Alsherif, E.A. |
| *Portulaca pilosa* L. | Wadi ELKor, 2019, 10085 (TUH) Fadl, M.A. & Alsherif, E.A. |
| *Psiadia punctulata* (DC.) Vatke | Wadi ELKor, 2019, 10032 (TUH) Fadl, M.A. & Alsherif, E.A. |
| *Pulicaria crispa* (Forssk.) Oliv. | Wadi ELKor, 2019, 10033 (TUH) Fadl, M.A. & Alsherif, E.A. |
| *Pulicaria guestii* Rech.f. & Rawi | Wadi ELKor, 2020, 10253 (TUH) Fadl, M.A. & Alsherif, E.A. |
| *Pulicaria incisa* (Lam.) DC. | Wadi ELKor, 2020, 10254 (TUH) Fadl, M.A. & Alsherif, E.A. |
| *Pulicaria inuloides* (Poir) DC. | Wadi ELKor, 2020, 10255 (TUH) Fadl, M.A. & Alsherif, E.A. |
| *Pulicaria schimperi* DC. | Wadi ELKor, 2020, 10256 (TUH) Fadl, M.A. & Alsherif, E.A. |
| *Pulicaria vulgaris* Gaertn. | Wadi ELKor, 2020, 10257 (TUH) Fadl, M.A. & Alsherif, E.A. |
| *Pupalia lappaceae* (Moq.) Hook. f. | Wadi ELKor, 2020, 10204 (TUH) Fadl, M.A. & Alsherif, E.A. |
| *Ricinus communis* L. | Wadi ELKor, 2019, 10042 (TUH) Fadl, M.A. & Alsherif, E.A. |
| *Rumex vesicarius* L. | Wadi ELKor, 2019, 10083 (TUH) Fadl, M.A. & Alsherif, E.A. |
| *Salsola imbricata* Forssk. | Wadi ELKor, 2020, 10247 (TUH) Fadl, M.A. & Alsherif, E.A. |
| *Salvia aegyptiaca* L. | Wadi ELKor, 2019, 10060 (TUH) Fadl, M.A. & Alsherif, E.A. |
| *Salvia lanigera* Poir. | Wadi ELKor, 2019, 10061 (TUH) Fadl, M.A. & Alsherif, E.A. |
| *Sclerocephalus arabicus* Boiss. | Wadi ELKor, 2020, 10241(TUH) Fadl, M.A. & Alsherif, E.A. |
| *Scorzonera tortuosissima* Boiss. | Wadi ELKor, 2020, 10258 (TUH) Fadl, M.A. & Alsherif, E.A. |
| *Scrophularia arguta* Sol. ex Ait | Wadi ELKor, 2019, 10099 (TUH) Fadl, M.A. & Alsherif, E.A. |
| *Seddera arabica* (Forssk.) Choisy | Wadi ELKor, 2020, 10264 (TUH) Fadl, M.A. & Alsherif, E.A. |
| *Seddera latifolia* Hochst. & Steud. | Wadi ELKor, 2020, 10265 (TUH) Fadl, M.A. & Alsherif, E.A. |
| *Senecio hoggariensis* Batt. & Trab. | Wadi ELKor, 2020, 10259 (TUH) Fadl, M.A. & Alsherif, E.A. |
| *Setaria viridis* (L.) P. Beauv. | Wadi ELKor, 2020, 10288 (TUH) Fadl, M.A. & Alsherif, E.A. |
| *Sidda alba* L. | Wadi ELKor, 2020, 10305 (TUH) Fadl, M.A. & Alsherif, E.A. |
| *Sisymbrium irio* L. | Wadi ELKor, 2020, 10270 (TUH) Fadl, M.A. & Alsherif, E.A. |
| *Solanum cordatum* Forssk. | Wadi ELKor, 2019, 10104 (TUH) Fadl, M.A. & Alsherif, E.A. |
| *Solanum forsskaolii* Dun. | Wadi ELKor, 2020, 10313 (TUH) Fadl, M.A. & Alsherif, E.A. |
| *Solanum glabratum* var. *sepicula* (Dunal) J.R.I. Wood | Wadi ELKor, 2020, 10317 (TUH) Fadl, M.A. & Alsherif, E.A. |
| *Solanum incanum* L. | Wadi ELKor, 2020, 10318 (TUH) Fadl, M.A. & Alsherif, E.A. |
| *Solanum nigrum* L. | Wadi ELKor, 2020, 10320 (TUH) Fadl, M.A. & Alsherif, E.A. |
| *Solanum villosum* Mill. | Wadi ELKor, 2020, 10321 (TUH) Fadl, M.A. & Alsherif, E.A. |
| *Sonchus oleraceus* L. | Wadi ELKor, 2020, 10260 (TUH) Fadl, M.A. & Alsherif, E.A. |
| *Spergula fallax* (Lowe) Krause | Wadi ELKor, 2020, 10242 (TUH) Fadl, M.A. & Alsherif, E.A. |
| *Stellaria pallida* (Dumort.) Pire | Wadi ELKor, 2020, 10243 (TUH) Fadl, M.A. & Alsherif, E.A. |
| *Stipa parviflora* Desf. | Wadi ELKor, 2020, 10290 (TUH) Fadl, M.A. & Alsherif, E.A. |
| *Stipagrostis obtusa* (Del.) Nees | Wadi ELKor, 2019, 10051 (TUH) Fadl, M.A. & Alsherif, E.A. |
| *Tamarix nilotica* (Ehrenb.) Bunge | Wadi ELKor, 2020, 10322 (TUH) Fadl, M.A. & Alsherif, E.A. |
| *Tephrosia nubica* (Boiss.) Bak. | Wadi ELKor, 2019, 10063 (TUH) Fadl, M.A. & Alsherif, E.A. |
| *Tephrosia purpuria* (L.) Pers. | Wadi ELKor, 2019, 10064 (TUH) Fadl, M.A. & Alsherif, E.A. |
| *Tetrapogon villosus* | Wadi ELKor, 2019, 10052 (TUH) Fadl, M.A. & Alsherif, E.A. |
| *Tetrapogon villosus* Desf. | Wadi ELKor, 2019, 10053 (TUH) Fadl, M.A. & Alsherif, E.A. |
| *Tribulus macropterus* Boiss. | Wadi ELKor, 2019, 10096 (TUH) Fadl, M.A. & Alsherif, E.A. |
| *Tribulus pentandrus* Forssk. | Wadi ELKor, 2019, 10097 (TUH) Fadl, M.A. & Alsherif, E.A. |
| *Tribulus terrestris* L. | Wadi ELKor, 2019, 10098 (TUH) Fadl, M.A. & Alsherif, E.A. |
| *Trichodesma africanum var.Africanum* (L.) R.Br. | Wadi ELKor, 2019, 10019 (TUH) Fadl, M.A. & Alsherif, E.A. |
| *Trichodesma trichodesmoides* (Bge.) Gürke | Wadi ELKor, 2019, 10020 (TUH) Fadl, M.A. & Alsherif, E.A. |
| *Triumfetta flavescens* Hochst. ex A. Rich. | Wadi ELKor, 2020, 10307 (TUH) Fadl, M.A. & Alsherif, E.A. |
| *Typha domingensis* (Pers.) Poir | Wadi ELKor, 2020, 10323 (TUH) Fadl, M.A. & Alsherif, E.A. |
| *Uritica urens* L. | Wadi ELKor, 2019, 10092 (TUH) Fadl, M.A. & Alsherif, E.A. |
| *Veronica anagallis-aquatica* L. | Wadi ELKor, 2019, 10100 (TUH) Fadl, M.A. & Alsherif, E.A. |
| *Zalyea pentandra* (L.) Jeffrey | Wadi ELKor, 2019, 10004 (TUH) Fadl, M.A. & Alsherif, E.A. |
| *Ziziphus spina-christi* (L.) Desf. | Wadi ELKor, 2019, 10087 (TUH) Fadl, M.A. & Alsherif, E.A. |
