# Supplementary material for: Impact of elevation and slope aspect on floristic composition in wadi Elkor, Sarawat Mountain, Saudi Arabia
Source: Sci Rep. 2021 Aug 9;11:16160. doi: 10.1038/s41598-021-95450-4 (PMC8352965; doi:10.1038/s41598-021-95450-4)
Supplement: Supplementary file 2 — Supplementary Information 2. [file 41598_2021_95450_MOESM2_ESM.docx]

Appendix 2: List of plant species recorded in the studied area with their families, life forms and chorotypes at different elevation sites. 1: present, 0: Absent, n: present in north face, s: present in south face, +: present in both faces.

| **Family** | **Species name** | **Site 1** | **Site 2.** | | | **Site 3** | **Life fom** | **Chorology** |
| --- | --- | --- | --- | --- | --- | --- | --- | --- |
|  |  |  | **1060** | **1160** | **1240** |  |  |  |
| *Acanthaceae* | *Blepharis ciliaris* (L.) B.L. Burtt | 1 | + | 0 | + | 1 | Ch | Sa-Si+S-Z |
|  | *Ecbolium viride* Alston | 0 | n | 0 | s | 0 | Ch | Sa-Si+S-Z |
|  | *Justicia flava* (Vahl) Vahl | 0 | + | + | 0 | 0 | Th | Sa-Si |
|  | *Peristrophe paniculata*(Forssk.) Brummit | 1 | 0 | 0 | 0 | 0 | Th | Palaeo |
| *Pteridaceae* | *Anogramma leptophylla* (L.) Link | 0 | n | 0 | 0 | 0 | Th | Cos |
| *Aizoaceae* | *Aizoon canarense* L. | 1 | + | + | + | 1 | Th | Sa-Si+S-Z |
|  | *Zalyea pentandra* (L.) Jeffrey | 1 | + | + | s | 0 | He | Sa-Si+M+I-T+S-Z |
| *Amaranthaceae* | *Achyranthes aspera* L. | 1 | n | + | + | 1 | Th | M+I-T |
|  | *Aerva javanica* (Burm.f.) Juss. ex Schultes | 1 | + | + | + | 1 | Ch | Sa-Si+S-Z |
|  | *Aerva lanata* (L.) Juss. | 1 | + | 0 | + | 0 | Ch | Sa-Si+S-Z |
|  | *Amaranthus cruentus* L. | 0 | 0 | 0 | 0 | 1 | Th | Pant + pal |
|  | *Amaranthus graecizans* L. | 1 | n | + | 0 | 0 | Th | Pant |
|  | *Amaranthus hybridus* L. | 1 | 0 | 0 | 0 | 0 | Th | Pant |
|  | *Amaranthus viridis* L. | 1 | 0 | 0 | 0 | 0 | Th | Pant |
|  | *Amaranthus lividus* L. | 0 | 0 | 0 | 0 | 0 | Th | Pant |
|  | *Digera muricata* (L.) Mart. | 0 | n | 0 | 0 | 0 | Th | Paleotrop |
|  | *Pupalia lappaceae* (Moq.) Hook. f. | 1 | + | n | + | 0 | Ch | Palaeo |
| *Asclepiadaceae* | *Calotropis procera* (Ait.) Ait. f. | 1 | + | n | s | 1 | Ch | Sa-Si+S-Z |
|  | *Caralluma retrospiciens* (Ehrenb.) N.E. Br. | 0 | + | + | + | 1 | Th | Sa-Si+S-Z |
|  | *Cynanchum acutum* L. | 0 | n | 0 | 0 | 0 | He | M + I-T+ Eur-Sib |
|  | *Glossonema boveanum* (Decne) Decne | 0 | 0 | n | 0 | 0 | Ch | S-Z |
|  | *Leptadenia pyrotechnica* (Forssk.) Decne | 1 | 0 | n | 0 | 0 | Ph | Sa-Si+S-Z |
|  | *Pergularia daemia* (Forssk.) Chiov. | 0 | + | 0 | s | 1 | Ch | Sa-Si+S-Z |
|  | *Pergularia tomentosa* L. | 0 | + | 0 | 0 | 0 | Ch | Sa-Si+S-Z |
| *Asparagaceae* | *Asparagus africanus* Lam. | 0 | n | n | n | 0 | Ch | Sa-Si+S-Z |
| *Asphodelaceae* | *Asphodelus tenuifolius* | *0* | 0 | 0 | 0 | 0 | Th | Sa-Si+M+I-T |
| *Boraginaceae* | *Anchusa milleri* Willd. | 0 | 0 | 0 | n | 0 | Th | Sa-Si+M |
|  | *Echium longiflorum* Del. | 0 | n | + | 0 | 0 | Th | Sa-Si |
|  | *Heliotropium arbainense* Fresen. | 0 | n | 0 | 0 | 1 | Ch | Sa-Si+S-Z |
|  | *Heliotropium longiflorum* (Hochst. & Steud.) Jaub. & Spach. | 0 | + | n | n | 0 | Ch | Sa-Si+S-Z |
|  | *Heliotropium strigosum* Willd. | 0 | 0 | s | 0 | 0 | Ch | Sa-Si+S-Z |
|  | *Heliotropium subulatumn* (DC.) Vatke | 0 | + | n | 0 | 0 | Ch | Sa-Si+S-Z |
|  | *Trichodesma africanum var.Africanum* (L.) R.Br. | 1 | n | 0 | 0 | 0 | Ch | Sa-Si+S-Z |
|  | *Trichodesma trichodesmoides* (Bge.) Gürke | 1 | 0 | 0 | 0 | 1 | Ch | Sa-Si+S-Z |
| *Bursuraceae* | *Commiphora kataf* Engl. | 1 | 0 | s | 0 | 0 | Ph | Sa-Si+S-Z |
|  | *Commiphora habissinica* (O. Berg.) Engl. | 0 | + | 0 | + | 0 | Ph | Sa-Si+S-Z |
|  | *Commiphora myrrha* Engl. | 1 | n | s | s | 0 | Ph | Sa-Si+S-Z |
| *Capparaceae* | *Cadaba farinose* Forssk. | 0 | 0 | 0 | n | 0 | Ph | Sa-Si+S-Z |
|  | *Capparis spinosa* L. | 0 | + | 0 | 0 | 0 | Ph | Sa-Si+M+I-T |
| *Caryophyllaceae* | *Cometes abyssinica* R.Br. | 0 | + | s | 0 | 0 | He | Sa-Si+S-Z |
|  | *Sclerocephalus arabicus* Boiss. | 0 | n | 0 | n | 0 | Th | Sa-Si+M |
|  | *Spergula fallax* (Lowe) Krause | 0 | + | 0 | 0 | 0 | Th | Sa-Si+M |
|  | *Stellaria pallida* (Dumort.) Pire | 1 | 0 | 0 | 0 | 0 | Th | M+Eur-Sib |
|  | *Chenopodium carinatum* R.Br. | 0 | 0 | 0 | 0 | 1 | Th | Cos |
| *Chenopodiaceae* | *Chenopodium murale* L. | 1 | + | + | + | 1 | Th | Cos |
|  | *Chenopodium opulifolium* Schrader ex Koch & Ziz | 0 | + | 0 | 0 | 0 | Th | Cos |
|  | *Salsola imbricata* Forssk. | 0 | 0 | 0 | 0 | 1 | ph | S-Z |
| *Cleomaceae* | *Cleome gynandra* L. | 0 | 0 | n | 0 | 0 | Th | Palaeo |
|  | *Cleome hanburyana* Penz. | 1 | 0 | 0 | 0 | 0 | Th | Sa-Si+S-Z |
|  | *Cleome ramosissima* Webb. | 0 | n | 0 | 0 | 0 | He | Sa-Si+S-Z |
|  | *Cleome scaposa* DC. | 1 | 0 | 0 | 0 | 0 | Th | Sa-Si+S-Z |
| *Commelinaceae* | *Commelina benghalensis* L. | 0 | n | 0 | + | 0 | G | Sa-Si+S-Z |
| *Compositae* | *Bidens biternata* (Lour) Merr. & Sherrf | 0 | n | n | 0 | 0 | Th | Pant |
|  | *Conyza bonariensis* (L.) Cronq. | 0 | n | 0 | 0 | 0 | Th | Pant |
|  | *Echinops hystrichoides* Kit-Tan | 0 | 0 | 0 | 0 | 1 | He | Sa-Si+S-Z |
|  | *Osteospermum vaillantii* (Decne.) Norlindh | 0 | n | n | 0 | 1 | Th | Sa-Si+S-Z |
|  | *Pluchea dioscoridis* (L.) DC. | 0 | 0 | 0 | 0 | 0 | Th | Palaeo |
|  | *Psiadia punctulata* (DC.) Vatke | 0 | + | 0 | 0 | 1 | Ch | Sa-Si+S-Z |
|  | *Pulicaria crispa* (Forssk.) Oliv. | 0 | n | 0 | 0 | 0 | Ch | Sa-Si+S-Z |
|  | *Pulicaria guestii* Rech.f. & Rawi | 0 | 0 | 0 | n | 0 | He | Sa-Si+S-Z |
|  | *Pulicaria incisa* (Lam.) DC. | 0 | n | 0 | 0 | 0 | Th | Sa-Si+S-Z |
|  | *Pulicaria inuloides* (Poir) DC. | 0 | + | n | s | 0 | Th | Sa-Si+S-Z |
|  | *Pulicaria schimperi* DC. | 1 | 0 | n | s | 0 | Th | Sa-Si |
|  | *Pulicaria vulgaris* Gaertn. | 0 | n | 0 | 0 | 0 | Th | Sa-Si+Med |
|  | *Scorzonera tortuosissima* Boiss. | 0 | 0 | 0 | 0 | 1 | Th |  |
|  | *Senecio hoggariensis* Batt. & Trab. | 0 | n | 0 | 0 | 0 | Th | Sa-Si |
|  | *Sonchus oleraceus* L. | 1 | n | n | n | 0 | Th | Cos |
| *Convolvulaceae* | *Convolvulus arvensis* L. | 0 | 0 | n | 0 | 0 | Ge | Cos |
|  | *Seddera arabica* (Forssk.) Choisy | 0 | 0 | n | 0 | 0 | He | Sa-Si+S-Z |
|  | *Seddera latifolia* Hochst. & Steud. | 0 | 0 | 0 | n | 0 | He | Sa-Si+S-Z |
| *Cruciferae* | *Farsetia longisiliqua* Decne. | 0 | 0 | 0 | s | 0 | Ch | Sa-Si+S-Z |
|  | *Morettia canescens* Boiss. | 1 | + | + | + | 0 | Th | Sa-Si |
|  | *Morettia parviflora* Boiss. | 1 | 0 | 0 | 0 | 0 | Th | Sa-Si |
|  | *Sisymbrium irio* L. | 0 | n | n | + | 1 | Th | Med +I-T + Sa-Si |
| *Cucurbitaceae* | *Coccinea grandis* (L.) Voigt. | 1 | n | + | + | 0 | He | Palaeo.+Pant. |
|  | *Citrullus colocynthis* (L.) Schrader | 1 | n | 0 | 0 | 0 | He | Sa-Si+M |
|  | *Citrullus lanatus* (Thunb.) Matsumara & Nakai | 0 | n | 0 | 0 | 0 | Th | Cult. |
|  | *Cucumis prophetarum var. prophetarum* L. | 0 | + | n | s | 0 | He | Sa-Si+S-Z |
| *Cyperaceae* | *Fimbristylis turkistanica* (Regel) B. Fedtsch. | 0 | s | 0 | 0 | 0 | Ge | Palaeo.+Pant. |
| *Ephedraceae* | *Ephedra foliate* Boiss. ex C.A. May | 0 | + | n | 0 | 0 | Ch | Sa-Si+I-T |
| *Euphorbiaceae* | *Acalypha fruticosa* Forssk. | 0 | n | + | 0 | 0 | Ph | Sa-Si+S-Z |
|  | *Chrozophora oblongifolia* (Del.) A. Juss. ex Spreng | 0 | n | 0 | 0 | 0 | Ch | M+I-T |
|  | *Euphorbia arabica* Hochst. & Wteyd | 1 | 0 | 0 | 0 | 0 | He | Sa-Si+M+S-Z |
|  | *Euphorbia granulata* Forssk. | 1 | n | 0 | 0 | 0 | Th | S-Z |
|  | *Euphorbia prostrata* Aiton | 0 | n | 0 | 0 | 0 | Th | Pant. |
|  | *Euphorbia schimperi* Presl. | 0 | n | 0 | n | 0 | Ch | Sa-Si |
|  | *Euphorbia serpens* Kunth | 0 | n | 0 | 0 | 0 | Th | Pant. |
|  | *Phyllanthus rotundifolius* | *0* | n | 0 | 0 | 0 | Th | Sa-Si+S-Z |
|  | *Phyllanthus tenellus* Roxb. | 1 | 0 | 0 | 0 | 0 | Th | Sa-Si+S-Z |
|  | *Ricinus communis* L. | 1 | 0 | 0 | 0 | 0 | Th | Sa-Si + S-Z |
| *Gramineae* | *Andropogon distachyos* L. | 0 | n | 0 | s | 1 | Ge | Palaeo + M |
|  | *Aristida mutabilis* Trin. & Rupr. | 0 | n | 0 | 0 | 0 | Th | Palaeo |
|  | *Arthraxon prionodes* (Steudel) Dandy | 0 | n | 0 | 0 | 0 | Th | Palaeo |
|  | *Brachiaria leersioides* (Hochst.) Stapf | 1 | n | n | 0 | 0 | Th | Palaeo |
|  | *Bromus diandrus* Roth | 0 | 0 | 0 | 0 | 1 | Th | ME + I-T |
|  | *Cenchrus ciliaris* L. | 1 | n | s | 0 | 0 | He | Palaeo |
|  | *Cenchrus pennisetiformis* Hochst. & Stedudel ex Steudel | 1 | + | + | + | 1 | Th | Sa-Si+I-T+S-Z |
|  | *Cenchrus longisetus ****M.C.Johnst. | 0 | n | 0 | 0 | 0 | He | Sa-Si+S-Z |
|  | *Cenchrus setigerus* Vahl | 1 | n | + | s | 0 | Ge | Sa-Si+S-Z |
|  | *Chrysopogon plumulosus* Hochst. | 0 | 0 | n | 0 | 0 | Ge | Palaeo |
|  | *Cynodon dactylon* (L.) Pers. | 1 | + | 0 | 0 | 1 | Ge | Sa-Si+S-Z+I-T |
|  | *Digitaria sanguinalis* (L.) Scop. | 0 | 0 | n | s | 0 | Th | Sa-Si+S-Z+I-T |
|  | *Echinochloa colona* (L.) Link | 1 | 0 | 0 | 0 | 0 | He | Palaeo |
|  | *Eragrostis pilosa* (L.) P. Beauv. | 0 | 0 | 0 | 0 | 1 | Th | Pal + Pant |
|  | *Panicum repens* L. | 0 | 0 | 0 | 0 | 1 | Th | Sa-Si+S-Z |
|  | *Eleusine indica ssp. Indica* (L.) Gaertn. | 1 | + | n | n | 0 | Th | Palaeo.+Pant. |
|  | *Pennisetum orientale* L.C. Rich | 0 | n | 0 | 0 | 1 | Ge | Sa-Si+M+I-T |
|  | *Phragmites australis* (Cav.) Trin. & Steudel. | 0 | n | 0 | 0 | 0 | G | Sa-Si+M+I-T |
|  | *Setaria viridis* (L.) P. Beauv. | 1 | n | n | 0 | 0 | Th | Sa-Si+M+I-T |
|  | *Stipa parviflora* Desf. | 1 | n | + | 0 | 0 | He | M+I-T |
|  | *Stipagrostis obtusa* (Del.) Nees | 0 | + | 0 | + | 0 | He | Sa-Si+S-Z |
|  | *Tetrapogon villosus* | 0 | + | + | + | 0 | Ge | Sa-Si+S-Z+I-T+M |
|  | *Tetrapogon villosus* Desf. | 1 | + | 0 | 0 | 0 | Ge | Sa-Si+S-Z+I-T+M |
| *Labiatae* | *Lavandula coronopifolia* Poir. | 0 | + | n | + | 1 | Ch | Sa-Si+S-Z+I-T |
|  | *Lavandula pubescens* Decne. | 0 | 0 | 0 | n | 0 | Ch | Sa-Si+S-Z |
|  | *Mentha longifolia ssp. schimperi* (Briq.) Briq. | 0 | s | 0 | 0 | 0 | Ge | I-T+M |
|  | *Ocimum forsskaolii* Benth. | 0 | + | + | + | 0 | Th | Sa-Si+S-Z |
|  | *Otostegia fruticosa ssp. schimperi* (Benth.) Sebald | 0 | + | + | + | 1 | Ch | Sa-Si |
|  | *Plectranthus tenuiflorus* (Vatke) Agnew | 0 | n | 0 | s | 0 | Ch | Sa-Si+S-Z |
|  | *Salvia aegyptiaca* L. | 0 | + | n | n | 0 | Ch | Sa-Si+S-Z |
|  | *Salvia lanigera* Poir. | 0 | 0 | 0 | n | 0 | Ch | Sa-Si+M |
| *Leguminosae* | *Acacia ehrenbergiana* Hayne | 1 | + | + | + | 0 | Ph | Sa-Si+S-Z |
|  | *Acacia etbaica* Schweinf. | 0 | + | 0 | 0 | 0 | Ph | Sa-Si+S-Z |
|  | *Acacia gerrardii* Benth. | 0 | + | 0 | 0 | 1 | Ph | Sa-Si+S-Z |
|  | *Acacia hamulosa* Benth. | 1 | + | + | + | 0 | Ph | Sa-Si+S-Z |
|  | *Acacia johnwoodii* Boulos | 1 | + | 0 | 0 | 0 | Ph | Sa-Si+S-Z |
|  | *Acacia tortilis* (Forssk.) Hayne | 1 | n | 0 | 0 | 0 | Ph | Sa-Si+S-Z |
|  | *Argyrolobium sp Nov.**** | 0 | n | 0 | 0 | 0 | Th | Sa-Si + I-T |
|  | *Cassia italic* | 0 | + | 0 | 0 | 0 | He | Sa-Si+S-Z |
|  | *Crotalaria emarginella* | 0 | + | 0 | + | 0 | Ch | Sa-Si+S-Z |
|  | *Crotalaria microphylla* | 0 | 0 | 0 | n | 0 | Th | Sa-Si+S-Z |
|  | *Indigofera articulata Nov****Gouan | 0 | 0 | 0 | 0 | 1 | Ch | Sa-Si+S-Z |
|  | *Indigofera hochstetteri* Bak*.* | 0 | n | n | 0 | 0 | Th | Sa-Si+S-Z |
|  | *Indigofera spinosa* Forssk*.* | 1 | n | + | + | 0 | Ch | Sa-Si+S-Z |
|  | *Indigofera trita ssp. subulata* | 0 | 0 | 0 | 0 | 0 | Ch | Paleo |
|  | *Tephrosia nubica* (Boiss.) Bak. | 1 | + | 0 | s | 0 | Ch | Sa-Si+S-Z |
|  | *Tephrosia purpuria* (L.) Pers. | 1 | n | + | + | 0 | Th | Sa-Si+S-Z |
| *Malvaceae* | *Abutilon hirtum* (Lamk.) Sweet. | 1 | n | n | 0 | 0 | Ch | Pant |
|  | *Abutilon bidentatum* A. Rich. | 1 | + | + | + | 1 | Ch | Sa-Si+S-Z |
|  | *Abutilon fruticosum* Guill. & Perr. | 0 | + | 0 | 0 | 0 | Ch | Sa-Si+S-Z |
|  | *Grewia erythraea* Schweinf. | 0 | n | 0 | s | 0 | Ch | Sa-Si+S-Z |
|  | *Grewia tenax* (Forssk.) Fiori | 1 | n | 0 | s | 0 | Ph | Sa-Si+S-Z |
|  | *Hibiscus micranthus* L. | 1 | 0 | 0 | 0 | 0 | Ch | Paleo |
|  | *Malva parviflora* L. | 1 | 0 | + | 0 | 1 | Th | M+I-T |
|  | *Melhania philippsiae* Baker | 0 | + | + | s | 0 | Ch | Sa-Si |
|  | *Sidda alba* L. | 1 | + | + | + | 0 | Ch | Sa-Si |
|  | *Triumfetta flavescens* Hochst. ex A. Rich. | 1 | + | + | + | 0 | Ch | Sa-Si+S-Z |
| *Menispermaceae* | *Coccolus pendulus* (J.R. & G. Forster) Diels | 1 | + | + | + | 0 | Ch | S-Z |
| *Molluginaceae* | *Corbichonia decumbens* (Forssk.) Exell | 1 | n | 0 | s | 0 | Th | Sa-Si+S-Z |
| *Moraceae* | *Ficus cordata ssp. salicifolia* (Vahl) C.C. Berg. | 0 | + | n | + | 0 | Ph | Sa-Si+S-Z |
|  | *Ficus palmata* Forssk. | 0 | + | 0 | 0 | 1 | Ph | Sa-Si+S-Z |
|  | *Ficus sycomorus* | 0 | s | s | 0 | 0 | Ph | Sa-Si+S-Z |
| *Nyctaginaceae* | *Boerhavia diffusa* L. | 0 | n | 0 | n | 0 | He | Pant |
|  | *Commicarpos ambiguus* Meikle | 0 | n | 0 | 0 | 0 | Ch | Sa-Si |
|  | *Commicarpos grandiflorus* (A. Rich.) Standley | 0 | n | n | 0 | 1 | Ch | Sa-Si+S-Z |
|  | *Commicarpos helenae* (Roem. & Schultes) Meikle | 1 | + | 0 | 0 | 0 | Ch | Sa-Si+I-T |
|  | *Commicarpos plumbagineus* (Cav.) Standley | 1 | + | 0 | + | 1 | He | Sa-Si+S-Z+M |
| *Palmae* | *Phoenix dactyliferae* | 0 | 0 | 0 | 0 | 0 | Ph | Sa-Si |
| *Papaveraceae* | *Argemone ochroleuca* Sweet. | 0 | + | 0 | 0 | 1 | Th | M+I-T |
| *Polygonaceae* | *Rumex vesicarius* L. | 0 | n | n | n | 0 | Th | Sa-Si |
| *Portulacaceae* | *Portulaca oleraceae* L. | 0 | n | n | + | 1 | Th | Cos |
|  | *Portulaca pilosa* L. | 0 | n | 0 | 0 | 0 | Th | Pant |
| *Resedaceae* | *Ochradenus baccatus* Del. | 1 | n | n | 0 | 1 | Ph | Sa-Si+S-Z |
| *Rhamnaceae* | *Ziziphus spina-christi* (L.) Desf. | 0 | n | 0 | s | 1 | Ph | Sa-Si+S-Z |
| *Scrophulariaceae* | *Anticharis arabica* Endl. | 0 | n | 0 | 0 | 0 | Th | Sa-Si |
|  | *Antirrhinum orontium* L. | 1 | n | 0 | 0 | 0 | Th | M |
|  | *Bacopa monnieri* (L.) Pennel. | 0 | s | 0 | 0 | 0 | He | Paleo |
|  | *Kickxia pseudoscoparia* V.W. Smith | 0 | n | 0 | n | 1 | Th | Endemic |
|  | *Lindenbergia indica var. indica* (L.) Vatke | 1 | n | s | 0 | 0 | Ch | Palaeo |
|  | *Scrophularia arguta* Sol. ex Ait | 1 | n | 0 | + | 0 | Th | Sa-Si+M |
|  | *Veronica anagallis-aquatica* L. | 0 | n | 0 | 0 | 0 | He | Cos |
| *Solanaceae* | *Datura innoxia* Mill. | 1 | + | n | 0 | 1 | Th | Pant |
|  | *Datura stramonium* L | 0 | n | 0 | 0 | 0 | Th | Pant |
|  | *Lycium shawii* Roem. & Schult. | 1 | n | + | + | 1 | Ph | Sa-Si+S-Z |
|  | *Solanum cordatum* Forssk. | 0 | n | n | n | 0 | Ch | Sa-Si |
|  | *Solanum forsskaolii* Dun. | 0 | + | n | n | 1 | Ch | Sa-Si |
|  | *Solanum glabratum* var. *sepicula* (Dunal) J.R.I. Wood | 0 | 0 | 0 | 0 | 1 | Th | Sa-Si + S-Z |
|  | *Solanum incanum* L. | 1 | + | n | 0 | 1 | Ch | Sa-Si |
|  | *Solanum nigrum* L. | 1 | 0 | 0 | 0 | 0 | Th | Cos |
|  | *Solanum villosum* Mill. | 0 | + | 0 | 0 | 1 | Th | M +I-T |
| *Tamaricaceae* | *Tamarix nilotica* (Ehrenb.) Bunge | 0 | s | 0 | 0 | 0 | Ph | Sa-Si+S-Z |
| *Typhaceae* | *Typha domingensis* (Pers.) Poir | 0 | s | 0 | 0 | 0 | Ge | Paleo. + Pant |
| *Umbelliferae* | *Apium nodiflorum* (L.) Lag. | 0 | 0 | n | 0 | 0 | He | Cos |
| *Urticaceae* | *Forsskaolea tenacissima* L. | 1 | + | + | + | 1 | He | Sa-Si+S-Z |
| *Verbinaceae* | *Uritica urens* L. | 0 | 0 | 0 | 0 | 1 | He | Sa-Si+S-Z |
| *Zygophyllaceae* | *Fagonia indica* Burm.f. | 0 | + | 0 | 0 | 1 | Ch | Sa-Si+I-T |
|  | *Fagonia paulayana* Wagner & Verh. | 1 | 0 | 0 | + | 0 | Ch | Sa-Si+I-T |
|  | *Peganum harmala* L. | 0 | 0 | 0 | 0 | 1 | Th | Sa-Si + I-T |
|  | *Tribulus macropterus* Boiss. | 1 | n | + | + | 0 | Th | Sa-Si+S-Z |
|  | *Tribulus pentandrus* Forssk. | 1 | 0 | 0 | 0 | 0 | Th | Sa-Si+S-Z |
|  | *Tribulus terrestris* L. | 1 | s | + | 0 | 1 | Th | Sa-Si+S-Z |
| *Acanthaceae* | *Blepharis ciliaris* (L.) B.L. Burtt | 1 | + | 0 | + | 1 | Ch | Sa-Si+S-Z |
|  | *Ecbolium viride* Alston | 0 | n | 0 | s | 0 | Ch | Sa-Si+S-Z |
|  | *Justicia flava* (Vahl) Vahl | 0 | + | + | 0 | 0 | Th | Sa-Si |
|  | *Peristrophe paniculata*(Forssk.) Brummit | 1 | 0 | 0 | 0 | 0 | Th | Palaeo |
| *Pteridaceae* | *Anogramma leptophylla* (L.) Link | 0 | n | 0 | 0 | 0 | Th | Cos |
| *Aizoaceae* | *Aizoon canarense* L. | 1 | + | + | + | 1 | Th | Sa-Si+S-Z |
|  | *Zalyea pentandra* (L.) Jeffrey | 1 | + | + | s | 0 | He | Sa-Si+M+I-T+S-Z |
| *Amaranthaceae* | *Achyranthes aspera* L. | 1 | n | + | + | 1 | Th | M+I-T |
|  | *Aerva javanica* (Burm.f.) Juss. ex Schultes | 1 | + | + | + | 1 | Ch | Sa-Si+S-Z |
|  | *Aerva lanata* (L.) Juss. | 1 | + | 0 | + | 0 | Ch | Sa-Si+S-Z |
|  | *Amaranthus cruentus* L. | 0 | 0 | 0 | 0 | 1 | Th | Pant + pal |
|  | *Amaranthus graecizans* L. | 1 | n | + | 0 | 0 | Th | Pant |
|  | *Amaranthus hybridus* L. | 1 | 0 | 0 | 0 | 0 | Th | Pant |
|  | *Amaranthus viridis* L. | 1 | 0 | 0 | 0 | 0 | Th | Pant |
|  | *Amaranthus lividus* L. | 0 | 0 | 0 | 0 | 0 | Th | Pant |
|  | *Digera muricata* (L.) Mart. | 0 | n | 0 | 0 | 0 | Th | Paleotrop |
|  | *Pupalia lappaceae* (Moq.) Hook. f. | 1 | + | n | + | 0 | Ch | Palaeo |
| *Asclepiadaceae* | *Calotropis procera* (Ait.) Ait. f. | 1 | + | n | s | 1 | Ch | Sa-Si+S-Z |
|  | *Caralluma retrospiciens* (Ehrenb.) N.E. Br. | 0 | + | + | + | 1 | Th | Sa-Si+S-Z |
|  | *Cynanchum acutum* L. | 0 | n | 0 | 0 | 0 | He | M + I-T+ Eur-Sib |
|  | *Glossonema boveanum* (Decne) Decne | 0 | 0 | n | 0 | 0 | Ch | S-Z |
|  | *Leptadenia pyrotechnica* (Forssk.) Decne | 1 | 0 | n | 0 | 0 | Ph | Sa-Si+S-Z |
|  | *Pergularia daemia* (Forssk.) Chiov. | 0 | + | 0 | s | 1 | Ch | Sa-Si+S-Z |
|  | *Pergularia tomentosa* L. | 0 | + | 0 | 0 | 0 | Ch | Sa-Si+S-Z |
| *Asparagaceae* | *Asparagus africanus* Lam. | 0 | n | n | n | 0 | Ch | Sa-Si+S-Z |
| *Asphodelaceae* | *Asphodelus tenuifolius* | *0* | 0 | 0 | 0 | 0 | Th | Sa-Si+M+I-T |
| *Boraginaceae* | *Anchusa milleri* Willd. | 0 | 0 | 0 | n | 0 | Th | Sa-Si+M |
|  | *Echium longiflorum* Del. | 0 | n | + | 0 | 0 | Th | Sa-Si |
|  | *Heliotropium arbainense* Fresen. | 0 | n | 0 | 0 | 1 | Ch | Sa-Si+S-Z |
|  | *Heliotropium longiflorum* (Hochst. & Steud.) Jaub. & Spach. | 0 | + | n | n | 0 | Ch | Sa-Si+S-Z |
|  | *Heliotropium strigosum* Willd. | 0 | 0 | s | 0 | 0 | Ch | Sa-Si+S-Z |
|  | *Heliotropium subulatumn* (DC.) Vatke | 0 | + | n | 0 | 0 | Ch | Sa-Si+S-Z |
|  | *Trichodesma africanum var.Africanum* (L.) R.Br. | 1 | n | 0 | 0 | 0 | Ch | Sa-Si+S-Z |
|  | *Trichodesma trichodesmoides* (Bge.) Gürke | 1 | 0 | 0 | 0 | 1 | Ch | Sa-Si+S-Z |
| *Bursuraceae* | *Commiphora kataf* Engl. | 1 | 0 | s | 0 | 0 | Ph | Sa-Si+S-Z |
|  | *Commiphora habissinica* (O. Berg.) Engl. | 0 | + | 0 | + | 0 | Ph | Sa-Si+S-Z |
|  | *Commiphora myrrha* Engl. | 1 | n | s | s | 0 | Ph | Sa-Si+S-Z |
| *Capparaceae* | *Cadaba farinose* Forssk. | 0 | 0 | 0 | n | 0 | Ph | Sa-Si+S-Z |
|  | *Capparis spinosa* L. | 0 | + | 0 | 0 | 0 | Ph | Sa-Si+M+I-T |
| *Caryophyllaceae* | *Cometes abyssinica* R.Br. | 0 | + | s | 0 | 0 | He | Sa-Si+S-Z |
|  | *Sclerocephalus arabicus* Boiss. | 0 | n | 0 | n | 0 | Th | Sa-Si+M |
|  | *Spergula fallax* (Lowe) Krause | 0 | + | 0 | 0 | 0 | Th | Sa-Si+M |
|  | *Stellaria pallida* (Dumort.) Pire | 1 | 0 | 0 | 0 | 0 | Th | M+Eur-Sib |
|  | *Chenopodium carinatum* R.Br. | 0 | 0 | 0 | 0 | 1 | Th | Cos |
| *Chenopodiaceae* | *Chenopodium murale* L. | 1 | + | + | + | 1 | Th | Cos |
|  | *Chenopodium opulifolium* Schrader ex Koch & Ziz | 0 | + | 0 | 0 | 0 | Th | Cos |
|  | *Salsola imbricata* Forssk. | 0 | 0 | 0 | 0 | 1 | ph | S-Z |
| *Cleomaceae* | *Cleome gynandra* L. | 0 | 0 | n | 0 | 0 | Th | Palaeo |
|  | *Cleome hanburyana* Penz. | 1 | 0 | 0 | 0 | 0 | Th | Sa-Si+S-Z |
|  | *Cleome ramosissima* Webb. | 0 | n | 0 | 0 | 0 | He | Sa-Si+S-Z |
|  | *Cleome scaposa* DC. | 1 | 0 | 0 | 0 | 0 | Th | Sa-Si+S-Z |
| *Commelinaceae* | *Commelina benghalensis* L. | 0 | n | 0 | + | 0 | G | Sa-Si+S-Z |
| *Compositae* | *Bidens biternata* (Lour) Merr. & Sherrf | 0 | n | n | 0 | 0 | Th | Pant |
|  | *Conyza bonariensis* (L.) Cronq. | 0 | n | 0 | 0 | 0 | Th | Pant |
|  | *Echinops hystrichoides* Kit-Tan | 0 | 0 | 0 | 0 | 1 | He | Sa-Si+S-Z |
|  | *Osteospermum vaillantii* (Decne.) Norlindh | 0 | n | n | 0 | 1 | Th | Sa-Si+S-Z |
|  | *Pluchea dioscoridis* (L.) DC. | 0 | 0 | 0 | 0 | 0 | Th | Palaeo |
|  | *Psiadia punctulata* (DC.) Vatke | 0 | + | 0 | 0 | 1 | Ch | Sa-Si+S-Z |
|  | *Pulicaria crispa* (Forssk.) Oliv. | 0 | n | 0 | 0 | 0 | Ch | Sa-Si+S-Z |
|  | *Pulicaria guestii* Rech.f. & Rawi | 0 | 0 | 0 | n | 0 | He | Sa-Si+S-Z |
|  | *Pulicaria incisa* (Lam.) DC. | 0 | n | 0 | 0 | 0 | Th | Sa-Si+S-Z |
|  | *Pulicaria inuloides* (Poir) DC. | 0 | + | n | s | 0 | Th | Sa-Si+S-Z |
|  | *Pulicaria schimperi* DC. | 1 | 0 | n | s | 0 | Th | Sa-Si |
|  | *Pulicaria vulgaris* Gaertn. | 0 | n | 0 | 0 | 0 | Th | Sa-Si+Med |
|  | *Scorzonera tortuosissima* Boiss. | 0 | 0 | 0 | 0 | 1 | Th |  |
|  | *Senecio hoggariensis* Batt. & Trab. | 0 | n | 0 | 0 | 0 | Th | Sa-Si |
|  | *Sonchus oleraceus* L. | 1 | n | n | n | 0 | Th | Cos |
| *Convolvulaceae* | *Convolvulus arvensis* L. | 0 | 0 | n | 0 | 0 | Ge | Cos |
|  | *Seddera arabica* (Forssk.) Choisy | 0 | 0 | n | 0 | 0 | He | Sa-Si+S-Z |
|  | *Seddera latifolia* Hochst. & Steud. | 0 | 0 | 0 | n | 0 | He | Sa-Si+S-Z |
| *Cruciferae* | *Farsetia longisiliqua* Decne. | 0 | 0 | 0 | s | 0 | Ch | Sa-Si+S-Z |
|  | *Morettia canescens* Boiss. | 1 | + | + | + | 0 | Th | Sa-Si |
|  | *Morettia parviflora* Boiss. | 1 | 0 | 0 | 0 | 0 | Th | Sa-Si |
|  | *Sisymbrium irio* L. | 0 | n | n | + | 1 | Th | Med +I-T + Sa-Si |
| *Cucurbitaceae* | *Coccinea grandis* (L.) Voigt. | 1 | n | + | + | 0 | He | Palaeo.+Pant. |
|  | *Citrullus colocynthis* (L.) Schrader | 1 | n | 0 | 0 | 0 | He | Sa-Si+M |
|  | *Citrullus lanatus* (Thunb.) Matsumara & Nakai | 0 | n | 0 | 0 | 0 | Th | Cult. |
|  | *Cucumis prophetarum var. prophetarum* L. | 0 | + | n | s | 0 | He | Sa-Si+S-Z |
| *Cyperaceae* | *Fimbristylis turkistanica* (Regel) B. Fedtsch. | 0 | s | 0 | 0 | 0 | Ge | Palaeo.+Pant. |
| *Ephedraceae* | *Ephedra foliate* Boiss. ex C.A. May | 0 | + | n | 0 | 0 | Ch | Sa-Si+I-T |
| *Euphorbiaceae* | *Acalypha fruticosa* Forssk. | 0 | n | + | 0 | 0 | Ph | Sa-Si+S-Z |
|  | *Chrozophora oblongifolia* (Del.) A. Juss. ex Spreng | 0 | n | 0 | 0 | 0 | Ch | M+I-T |
|  | *Euphorbia arabica* Hochst. & Wteyd | 1 | 0 | 0 | 0 | 0 | He | Sa-Si+M+S-Z |
|  | *Euphorbia granulata* Forssk. | 1 | n | 0 | 0 | 0 | Th | S-Z |
|  | *Euphorbia prostrata* Aiton | 0 | n | 0 | 0 | 0 | Th | Pant. |
|  | *Euphorbia schimperi* Presl. | 0 | n | 0 | n | 0 | Ch | Sa-Si |
|  | *Euphorbia serpens* Kunth | 0 | n | 0 | 0 | 0 | Th | Pant. |
|  | *Phyllanthus rotundifolius* | *0* | n | 0 | 0 | 0 | Th | Sa-Si+S-Z |
|  | *Phyllanthus tenellus* Roxb. | 1 | 0 | 0 | 0 | 0 | Th | Sa-Si+S-Z |
|  | *Ricinus communis* L. | 1 | 0 | 0 | 0 | 0 | Th | Sa-Si + S-Z |
| *Gramineae* | *Andropogon distachyos* L. | 0 | n | 0 | s | 1 | Ge | Palaeo + M |
|  | *Aristida mutabilis* Trin. & Rupr. | 0 | n | 0 | 0 | 0 | Th | Palaeo |
|  | *Arthraxon prionodes* (Steudel) Dandy | 0 | n | 0 | 0 | 0 | Th | Palaeo |
|  | *Brachiaria leersioides* (Hochst.) Stapf | 1 | n | n | 0 | 0 | Th | Palaeo |
|  | *Bromus diandrus* Roth | 0 | 0 | 0 | 0 | 1 | Th | ME + I-T |
|  | *Cenchrus ciliaris* L. | 1 | n | s | 0 | 0 | He | Palaeo |
|  | *Cenchrus pennisetiformis* Hochst. & Stedudel ex Steudel | 1 | + | + | + | 1 | Th | Sa-Si+I-T+S-Z |
|  | *Cenchrus longisetus ****M.C.Johnst. | 0 | n | 0 | 0 | 0 | He | Sa-Si+S-Z |
|  | *Cenchrus setigerus* Vahl | 1 | n | + | s | 0 | Ge | Sa-Si+S-Z |
|  | *Chrysopogon plumulosus* Hochst. | 0 | 0 | n | 0 | 0 | Ge | Palaeo |
|  | *Cynodon dactylon* (L.) Pers. | 1 | + | 0 | 0 | 1 | Ge | Sa-Si+S-Z+I-T |
|  | *Digitaria sanguinalis* (L.) Scop. | 0 | 0 | n | s | 0 | Th | Sa-Si+S-Z+I-T |
|  | *Echinochloa colona* (L.) Link | 1 | 0 | 0 | 0 | 0 | He | Palaeo |
|  | *Eragrostis pilosa* (L.) P. Beauv. | 0 | 0 | 0 | 0 | 1 | Th | Pal + Pant |
|  | *Panicum repens* L. | 0 | 0 | 0 | 0 | 1 | Th | Sa-Si+S-Z |
|  | *Eleusine indica ssp. Indica* (L.) Gaertn. | 1 | + | n | n | 0 | Th | Palaeo.+Pant. |
|  | *Pennisetum orientale* L.C. Rich | 0 | n | 0 | 0 | 1 | Ge | Sa-Si+M+I-T |
|  | *Phragmites australis* (Cav.) Trin. & Steudel. | 0 | n | 0 | 0 | 0 | G | Sa-Si+M+I-T |
|  | *Setaria viridis* (L.) P. Beauv. | 1 | n | n | 0 | 0 | Th | Sa-Si+M+I-T |
|  | *Stipa parviflora* Desf. | 1 | n | + | 0 | 0 | He | M+I-T |
|  | *Stipagrostis obtusa* (Del.) Nees | 0 | + | 0 | + | 0 | He | Sa-Si+S-Z |
|  | *Tetrapogon villosus* | 0 | + | + | + | 0 | Ge | Sa-Si+S-Z+I-T+M |
|  | *Tetrapogon villosus* Desf. | 1 | + | 0 | 0 | 0 | Ge | Sa-Si+S-Z+I-T+M |
| *Labiatae* | *Lavandula coronopifolia* Poir. | 0 | + | n | + | 1 | Ch | Sa-Si+S-Z+I-T |
|  | *Lavandula pubescens* Decne. | 0 | 0 | 0 | n | 0 | Ch | Sa-Si+S-Z |
|  | *Mentha longifolia ssp. schimperi* (Briq.) Briq. | 0 | s | 0 | 0 | 0 | Ge | I-T+M |
|  | *Ocimum forsskaolii* Benth. | 0 | + | + | + | 0 | Th | Sa-Si+S-Z |
|  | *Otostegia fruticosa ssp. schimperi* (Benth.) Sebald | 0 | + | + | + | 1 | Ch | Sa-Si |
|  | *Plectranthus tenuiflorus* (Vatke) Agnew | 0 | n | 0 | s | 0 | Ch | Sa-Si+S-Z |
|  | *Salvia aegyptiaca* L. | 0 | + | n | n | 0 | Ch | Sa-Si+S-Z |
|  | *Salvia lanigera* Poir. | 0 | 0 | 0 | n | 0 | Ch | Sa-Si+M |
| *Leguminosae* | *Acacia ehrenbergiana* Hayne | 1 | + | + | + | 0 | Ph | Sa-Si+S-Z |
|  | *Acacia etbaica* Schweinf. | 0 | + | 0 | 0 | 0 | Ph | Sa-Si+S-Z |
|  | *Acacia gerrardii* Benth. | 0 | + | 0 | 0 | 1 | Ph | Sa-Si+S-Z |
|  | *Acacia hamulosa* Benth. | 1 | + | + | + | 0 | Ph | Sa-Si+S-Z |
|  | *Acacia johnwoodii* Boulos | 1 | + | 0 | 0 | 0 | Ph | Sa-Si+S-Z |
|  | *Acacia tortilis* (Forssk.) Hayne | 1 | n | 0 | 0 | 0 | Ph | Sa-Si+S-Z |
|  | *Argyrolobium sp Nov.**** | 0 | n | 0 | 0 | 0 | Th | Sa-Si + I-T |
|  | *Cassia italic* | 0 | + | 0 | 0 | 0 | He | Sa-Si+S-Z |
|  | *Crotalaria emarginella* | 0 | + | 0 | + | 0 | Ch | Sa-Si+S-Z |
|  | *Crotalaria microphylla* | 0 | 0 | 0 | n | 0 | Th | Sa-Si+S-Z |
|  | *Indigofera articulata Nov****Gouan | 0 | 0 | 0 | 0 | 1 | Ch | Sa-Si+S-Z |
|  | *Indigofera hochstetteri* Bak*.* | 0 | n | n | 0 | 0 | Th | Sa-Si+S-Z |
|  | *Indigofera spinosa* Forssk*.* | 1 | n | + | + | 0 | Ch | Sa-Si+S-Z |
|  | *Indigofera trita ssp. subulata* | 0 | 0 | 0 | 0 | 0 | Ch | Paleo |
|  | *Tephrosia nubica* (Boiss.) Bak. | 1 | + | 0 | s | 0 | Ch | Sa-Si+S-Z |
|  | *Tephrosia purpuria* (L.) Pers. | 1 | n | + | + | 0 | Th | Sa-Si+S-Z |
| *Malvaceae* | *Abutilon hirtum* (Lamk.) Sweet. | 1 | n | n | 0 | 0 | Ch | Pant |
|  | *Abutilon bidentatum* A. Rich. | 1 | + | + | + | 1 | Ch | Sa-Si+S-Z |
|  | *Abutilon fruticosum* Guill. & Perr. | 0 | + | 0 | 0 | 0 | Ch | Sa-Si+S-Z |
|  | *Grewia erythraea* Schweinf. | 0 | n | 0 | s | 0 | Ch | Sa-Si+S-Z |
|  | *Grewia tenax* (Forssk.) Fiori | 1 | n | 0 | s | 0 | Ph | Sa-Si+S-Z |
|  | *Hibiscus micranthus* L. | 1 | 0 | 0 | 0 | 0 | Ch | Paleo |
|  | *Malva parviflora* L. | 1 | 0 | + | 0 | 1 | Th | M+I-T |
|  | *Melhania philippsiae* Baker | 0 | + | + | s | 0 | Ch | Sa-Si |
|  | *Sidda alba* L. | 1 | + | + | + | 0 | Ch | Sa-Si |
|  | *Triumfetta flavescens* Hochst. ex A. Rich. | 1 | + | + | + | 0 | Ch | Sa-Si+S-Z |
| *Menispermaceae* | *Coccolus pendulus* (J.R. & G. Forster) Diels | 1 | + | + | + | 0 | Ch | S-Z |
| *Molluginaceae* | *Corbichonia decumbens* (Forssk.) Exell | 1 | n | 0 | s | 0 | Th | Sa-Si+S-Z |
| *Moraceae* | *Ficus cordata ssp. salicifolia* (Vahl) C.C. Berg. | 0 | + | n | + | 0 | Ph | Sa-Si+S-Z |
|  | *Ficus palmata* Forssk. | 0 | + | 0 | 0 | 1 | Ph | Sa-Si+S-Z |
|  | *Ficus sycomorus* | 0 | s | s | 0 | 0 | Ph | Sa-Si+S-Z |
| *Nyctaginaceae* | *Boerhavia diffusa* L. | 0 | n | 0 | n | 0 | He | Pant |
|  | *Commicarpos ambiguus* Meikle | 0 | n | 0 | 0 | 0 | Ch | Sa-Si |
|  | *Commicarpos grandiflorus* (A. Rich.) Standley | 0 | n | n | 0 | 1 | Ch | Sa-Si+S-Z |
|  | *Commicarpos helenae* (Roem. & Schultes) Meikle | 1 | + | 0 | 0 | 0 | Ch | Sa-Si+I-T |
|  | *Commicarpos plumbagineus* (Cav.) Standley | 1 | + | 0 | + | 1 | He | Sa-Si+S-Z+M |
| *Palmae* | *Phoenix dactyliferae* | 0 | 0 | 0 | 0 | 0 | Ph | Sa-Si |
| *Papaveraceae* | *Argemone ochroleuca* Sweet. | 0 | + | 0 | 0 | 1 | Th | M+I-T |
| *Polygonaceae* | *Rumex vesicarius* L. | 0 | n | n | n | 0 | Th | Sa-Si |
| *Portulacaceae* | *Portulaca oleraceae* L. | 0 | n | n | + | 1 | Th | Cos |
|  | *Portulaca pilosa* L. | 0 | n | 0 | 0 | 0 | Th | Pant |
| *Resedaceae* | *Ochradenus baccatus* Del. | 1 | n | n | 0 | 1 | Ph | Sa-Si+S-Z |
| *Rhamnaceae* | *Ziziphus spina-christi* (L.) Desf. | 0 | n | 0 | s | 1 | Ph | Sa-Si+S-Z |
| *Scrophulariaceae* | *Anticharis arabica* Endl. | 0 | n | 0 | 0 | 0 | Th | Sa-Si |
|  | *Antirrhinum orontium* L. | 1 | n | 0 | 0 | 0 | Th | M |
|  | *Bacopa monnieri* (L.) Pennel. | 0 | s | 0 | 0 | 0 | He | Paleo |
|  | *Kickxia pseudoscoparia* V.W. Smith | 0 | n | 0 | n | 1 | Th | Endemic |
|  | *Lindenbergia indica var. indica* (L.) Vatke | 1 | n | s | 0 | 0 | Ch | Palaeo |
|  | *Scrophularia arguta* Sol. ex Ait | 1 | n | 0 | + | 0 | Th | Sa-Si+M |
|  | *Veronica anagallis-aquatica* L. | 0 | n | 0 | 0 | 0 | He | Cos |
| *Solanaceae* | *Datura innoxia* Mill. | 1 | + | n | 0 | 1 | Th | Pant |
|  | *Datura stramonium* L | 0 | n | 0 | 0 | 0 | Th | Pant |
|  | *Lycium shawii* Roem. & Schult. | 1 | n | + | + | 1 | Ph | Sa-Si+S-Z |
|  | *Solanum cordatum* Forssk. | 0 | n | n | n | 0 | Ch | Sa-Si |
|  | *Solanum forsskaolii* Dun. | 0 | + | n | n | 1 | Ch | Sa-Si |
|  | *Solanum glabratum* var. *sepicula* (Dunal) J.R.I. Wood | 0 | 0 | 0 | 0 | 1 | Th | Sa-Si + S-Z |
|  | *Solanum incanum* L. | 1 | + | n | 0 | 1 | Ch | Sa-Si |
|  | *Solanum nigrum* L. | 1 | 0 | 0 | 0 | 0 | Th | Cos |
|  | *Solanum villosum* Mill. | 0 | + | 0 | 0 | 1 | Th | M +I-T |
| *Tamaricaceae* | *Tamarix nilotica* (Ehrenb.) Bunge | 0 | s | 0 | 0 | 0 | Ph | Sa-Si+S-Z |
| *Typhaceae* | *Typha domingensis* (Pers.) Poir | 0 | s | 0 | 0 | 0 | Ge | Paleo. + Pant |
| *Umbelliferae* | *Apium nodiflorum* (L.) Lag. | 0 | 0 | n | 0 | 0 | He | Cos |
| *Urticaceae* | *Forsskaolea tenacissima* L. | 1 | + | + | + | 1 | He | Sa-Si+S-Z |
| *Verbinaceae* | *Uritica urens* L. | 0 | 0 | 0 | 0 | 1 | He | Sa-Si+S-Z |
| *Zygophyllaceae* | *Fagonia indica* Burm.f. | 0 | + | 0 | 0 | 1 | Ch | Sa-Si+I-T |
|  | *Fagonia paulayana* Wagner & Verh. | 1 | 0 | 0 | + | 0 | Ch | Sa-Si+I-T |
|  | *Peganum harmala* L. | 0 | 0 | 0 | 0 | 1 | Th | Sa-Si + I-T |
|  | *Tribulus macropterus* Boiss. | 1 | n | + | + | 0 | Th | Sa-Si+S-Z |
|  | *Tribulus pentandrus* Forssk. | 1 | 0 | 0 | 0 | 0 | Th | Sa-Si+S-Z |
|  | *Tribulus terrestris* L. | 1 | s | + | 0 | 1 | Th | Sa-Si+S-Z |

The life forms are: Ph, phanerophytes; Ch, chamaephytes; G, geophytes; He, hemi-cryptophytes and Th, therophytes. The chorotypes are: COSM, cosmopolitan AM, American; I-T, Irano-Turanian; M, Mediterranean; SAa-Si Saharo-Sindian; S-Z, Sudano-Zambezian; Pal: Plaeotropical, Pant: Pantropic and TR, Tropical.
